# Supplementary material for: Quantum Mechanical Assessment of Protein–Ligand Hydrogen Bond Strength Patterns: Insights from Semiempirical Tight-Binding and Local Vibrational Mode Theory
Source: Int J Mol Sci. 2023 Mar 27;24(7):6311. doi: 10.3390/ijms24076311 (PMC10094336; doi:10.3390/ijms24076311)
Supplement: Supplementary file 1 [file ijms-24-06311-s001.zip › ijms-2283281-supplementary.pdf]

International Journal of
Molecular Sciences

MDPI

Article

Quantum Mechanical Assessment of Protein–Ligand Hydrogen Bond Strength Patterns: Insights from Semiempirical Tight-Binding and Local Vibrational Mode Theory

Ayesh Madushanka [1], Renaldo T. Moura, Jr. [2], Niraj Verma [1] and Elfi Kraka [1,*]

[1] Computational and Theoretical Chemistry Group (CATCO), Department of Chemistry, Southern Methodist University, 3215 Daniel Ave, Dallas, TX 75275-0314, USA
[2] Department of Chemistry and Physics, Center of Agrarian Sciences, Federal University of Paraiba, Areia 58397-000, Brazil
* Correspondence: ekraka@smu.edu

check for updates

Citation: Madushanka, A.; Moura, R.T., Jr.; Verma, N.; Kraka, E. Quantum Mechanical Assessment of Protein–Ligand Hydrogen Bond Strength Patterns: Insights from Semiempirical Tight-Binding and Local Vibrational Mode Theory. *Int. J. Mol. Sci.* **2023**, *24*, 6311. https://doi.org/10.3390/ijms24076311

Academic Editor: M. Natália D.S. Cordeiro

Received: 28 February 2023
Revised: 19 March 2023
Accepted: 23 March 2023
Published: 27 March 2023

Copyright: © 2023 by the authors. Licensee MDPI, Basel, Switzerland. This article is an open access article distributed under the terms and conditions of the Creative Commons Attribution (CC BY) license (https://creativecommons.org/licenses/by/4.0/).

Contents

1. To access PDB IDs 1
2. PDB IDs for database A 1
3. PDB IDs for database B 13

1. To access PDB IDs

All the experimental structures related to the PDB IDs mentioned below can be accessed through the RCSB data bank (https://www.rcsb.org/accessed on 15 October 2022).

2. PDB IDs for database A

1f92 1kdv 1p05 1utj 2am2 2gg7 2oc4 2uz9 2xp4 3bls 3f35 3ii5 3mpt 3qbh 3tdz 3zlk 4c4e 4fjz 4j17 4m3b 4p7m 10gs 1f9e 1kdw 1p06 1utl 2am4 2gg8 2oc7 2uzb 2xp5 3blt 3f36 3iit 3mqf 3qbn 3te5 3zll 4c4f 4fk6 4j1c 4m3d 4p7s 11gs 1f9g 1kdy 1p0y 1utm 2am9 2gg9 2oc9 2uzd 2xp6 3blu 3f37 3iiw 3mrt 3qc4 3tei 3zln 4c4g 4fk7 4j1f 4m3e 4pax 13gs 1fao 1kdz 1p10 1utn 2ama 2gga 2odd 2uze 2xp7 3bm6 3f38 3iiy 3mrv 3qc9 3tf6 3zlo 4c4h 4fkk 4j1h 4m3f 4pb1 16pk 1fax 1ke0 1p17 1uto 2amt 2ggb 2oei 2uzj 2xp8 3bm8 3f39 3ij0 3mrx 3qce 3tf7 3zlq 4c4i 4fl1 4j1i 4m3g 4pb2 184l 1fbm 1ke1 1p19 1utp 2amv 2ggd 2of2 2uzl 2xpa 3bm9 3f3a 3ij1 3ms2 3qcf 3tfk 3zlr 4c4j 4fl2 4j1k 4m3m 4pce 185l 1fch 1ke2 1p1n 1utr 2an5 2ggu 2of4 2uzn 2xpb 3bmn 3f3c 3ijg 3ms4 3qch 3tfn 3zls 4c4n 4fl3 4j1p 4m3p 4pci 186l 1fcx 1ke3 1p1o 1utt 2ank 2ggx 2off 2uzo 2xpc 3bmo 3f3d 3ijh 3ms7 3qci 3tfp 3zlv 4c52 4flh 4j21 4m3q 4pcs 187l 1fcz 1ke5 1p1q 1utz 2anl 2gh6 2ofu 2uzv 2xpk 3bmq 3f3e 3ijy 3ms9 3qcj 3tfu 3zlw 4c5d 4fli 4j22 4m48 4pd5 188l 1fd0 1ke6 1p28 1uu3 2anm 2gh7 2ofv 2v00 2xqq 3bmy 3f3t 3ijz 3msc 3qck 3tfv 3zlx 4c5w 4flj 4j24 4m4q 4pd6 1a07 1fd7 1ke7 1p2a 1uu7 2ans 2gh9 2og8 2v0c 2xru 3bpc 3f3u 3ik1 3msj 3qcl 3tg5 3zly 4c61 4flk 4j26 4m5g 4pd7 1a08 1fdq 1ke8 1p2g 1uu8 2ao6 2ghg 2ogy 2v0n 2xrw 3bpr 3f3v 3ik3 3msk 3qcq 3tge 3zm4 4c66 4fll 4j28 4m5h 4pd8 1a09 1fe3 1ke9 1p4r 1uu9 2aoc 2giu 2ogz 2v0z 2xs0 3bqc 3f3w 3ika 3msl 3qcs 3tgs 3zm5 4c68 4fm7 4j2c 4m5i 4pd9 1a0q 1fgi 1kel 1p4u 1uv5 2aod 2gj5 2oh0 2v10 2xs8 3bqn 3f48 3ikc 3mss 3qcx 3th0 3zm6 4c6u 4fm8 4j2t 4m5j 4pda 1a0t 1fh7 1kf0 1p57 1uv6 2aoe 2gkl 2oh4 2v11 2xsb 3br9 3f5j 3ikd 3mt7 3qcy 3th8 3zm9 4c6v 4fmn 4j3d 4m5l 4pee 1a1b 1fh8 1kf6 1p5e 1uvr 2aof 2gl0 2ohk 2v12 2xtk 3bra 3f5k 3ikg 3mt8 3qd0 3th9 3zmh 4c6x 4fmo 4j3e 4m5m 4pf3 1a1c 1fh9 1kfy 1p6d 1uvs 2aog 2glm 2ohl 2v13 2xtn 3brn 3f5l 3il5 3mt9 3qd3 3thb 3zmi 4c6z 4fmq 4j3i 4m5n 4pf5 1a1e 1fhd 1kjr 1p6e 1uvt 2aoh 2glp 2ohm 2v16 2xto 3bsc 3f5p 3il6 3mta 3qd4 3thd 3zmj 4c70 4fmu 4j3j 4m5o 4pft 1a28 1fhr 1kkq 1p7m 1uvu 2aoi 2gm1 2ohp 2v22 2xu1 3bt9 3f66 3ilq 3mtb 3qdd 3ti1 3zmp 4c71 4fnn 4j3l 4m5r 4pfu

1a2c 1fig 1kl3 1p93 1uw6 2aoj 2gmk 2ohq 2v25 2xu3 3btc 3f68 3imc 3mtd 3qel 3ti3 3zmq 4c72 4fns 4j3m 4m6p 4pg3 1a30 1fiv 1kl5 1pa9 1uwb 2aou 2gmv 2ohr 2v2c 2xu4 3bti 3f69 3ime 3mtw 3qem 3ti4 3zmt 4c73 4fny 4j44 4m6q 4pgh 1a3e 1fj4 1klg 1pau 1uwf 2aov 2gmx 2ohs 2v2h 2xu5 3btj 3f6e 3img 3muf 3qfd 3ti5 3zmu 4c7t 4fnz 4j45 4m6u 4ph4 1a42 1fjs 1kll 1pb8 1uwh 2aow 2gnf 2oht 2v2q 2xuc 3btl 3f6g 3imy 3muk 3qfv 3ti6 3zmv 4c8r 4fob 4j46 4m7b 4phu 1a46 1fkb 1klu 1pb9 1uwt 2aox 2gnh 2ohu 2v2v 2xuf 3btr 3f6h 3in3 3muz 3qfy 3ti8 3zmz 4c94 4foc 4j47 4m7c 4phv 1a4g 1fkf 1km3 1pbk 1uwu 2aq7 2gni 2ohv 2v3d 2xui 3bu1 3f70 3in4 3mv0 3qfz 3tia 3zn0 4c9w 4fod 4j48 4m7j 4phw 1a4h 1fkg 1kmv 1pbq 1ux7 2aq9 2gnj 2oi0 2v3e 2xup 3bu6 3f78 3ind 3mv5 3qg6 3tib 3zn1 4ca5 4fp1 4j4n 4m84 4pid 1a4k 1fkh 1kmy 1pcg 1uxa 2aqb 2gnl 2oi2 2v3u 2xuz 3bu8 3f7b 3ine 3mvh 3qgw 3tic 3znc 4ca6 4fpk 4j4o 4m8e 4pin 1a4m 1fki 1kna 1pdq 1uxb 2aqu 2go4 2oi3 2v54 2xv1 3buf 3f7g 3inf 3mvj 3qgy 3tif 3znr 4ca7 4fqo 4j4v 4m8h 4pio 1a4q 1fkn 1kne 1pdz 1uy6 2arm 2gph 2oi9 2v57 2xvd 3bug 3f7h 3inh 3mvl 3qi1 3tiw 3zns 4ca8 4fr3 4j51 4m8x 4piq 1a4r 1fkw 1koj 1pf7 1uy7 2asu 2gpp 2oic 2v58 2xvn 3buh 3f7i 3iny 3mvm 3qi2 3tiy 3zo1 4cae 4fri 4j52 4m8y 4pis 1a4w 1fl3 1kpm 1pf8 1uy8 2ate 2gqn 2oiq 2v59 2xwd 3bum 3f7u 3io7 3mw1 3qi3 3tiz 3zo2 4caf 4frj 4j53 4man 4pjt 1a50 1fl6 1kqb 1pfu 1uy9 2ath 2gss 2oj9 2v5a 2xwe 3bun 3f7z 3iob 3mwu 3qi4 3tjc 3zo4 4cbt 4frk 4j5p 4mb9 4pkr 1a52 1flm 1kr3 1pfy 1uyc 2auc 2gsu 2ojf 2v5x 2xwy 3buo 3f80 3ioc 3mww 3qin 3tjd 3zos 4cby 4frs 4j6i 4mbc 4pks 1a5g 1flr 1ksn 1pg2 1uyd 2aux 2gtk 2ojg 2v6n 2xx4 3buw 3f81 3iod 3mxc 3qio 3tjh 3zot 4cc2 4fs3 4j70 4mbf 4pkt 1a5h 1fls 1kti 1pgp 1uye 2auz 2gtv 2oji 2v77 2xx5 3bux 3f82 3ioe 3mxd 3qip 3tjm 3zov 4cc3 4fs4 4j73 4mbi 4pku 1a5v 1fm9 1ktt 1ph0 1uyf 2avi 2gu8 2ojj 2v7a 2xxn 3bv2 3f88 3iof 3mxe 3qiy 3tk2 3zp9 4cc5 4fse 4j74 4mbj 4pkv 1a61 1fmb 1kug 1phw 1uyg 2avm 2gv2 2ok1 2v7d 2xxr 3bv3 3f8c 3iog 3mxf 3qiz 3tkh 3zpq 4cc7 4fsl 4j77 4mbl 4pkw 1a69 1fo0 1kui 1pi4 1uyh 2avo 2gv7 2olb 2v83 2xxt 3bv9 3f8e 3ioi 3mxr 3qj0 3tki 3zpr 4ccd 4ft2 4j78 4mbp 4pl0 1a7c 1fo3 1kuk 1pig 1uyi 2avq 2gvd 2ole 2v85 2xxw 3bva 3f8f 3iok 3mxs 3qj9 3tkm 3zps 4cd0 4fut 4j79 4mc1 4pl3 1a7t 1fpc 1kv1 1pip 1uyk 2avs 2gvf 2on3 2v86 2xxx 3bvb 3f8s 3ion 3mxy 3qk0 3tku 3zpt 4cd4 4fvq 4j7d 4mc2 4pl4 1a7x 1fpi 1kv2 1pk0 1uym 2avv 2gvj 2on6 2v87 2xxy 3bwf 3f8w 3iop 3my1 3qk5 3tkw 3zpu 4cd5 4fvr 4j7e 4mc6 4pl5 1a85 1fpp 1kv5 1pkx 1uys 2aw1 2gvv 2onb 2v88 2xy9 3bwj 3f9n 3ip5 3my5 3qkk 3tkz 3zq9 4cd6 4fxf 4j7i 4mc9 4pm0 1a86 1fpy 1kvo 1pl0 1uz1 2ax6 2gvz 2onc 2v8w 2xyd 3bwk 3f9w 3ip6 3myg 3qkl 3tl0 3zqi 4cd8 4fxj 4j81 4mcb 4pml 1a8i 1fq4 1kwq 1pme 1uz4 2ax9 2gyi 2ony 2v96 2xye 3bx5 3f9y 3ip8 3myq 3qkm 3tl5 3zqt 4cdr 4fxp 4j82 4mcc 4pmm 1a8t 1fq5 1kwr 1pmn 1uz8 2axi 2gz2 2onz 2va5 2xyf 3bxe 3fa3 3ip9 3mz3 3qkv 3tlh 3zrc 4ce1 4fxq 4j84 4mcv 4pms 1a94 1fq6 1kyn 1pmq 1v0l 2ay1 2gz7 2oo8 2va6 2xyn 3bxf 3faa 3ipa 3mz6 3ql9 3tll 3zrk 4ce2 4fxy 4j86 4md6 4pmt 1a99 1fq7 1kyv 1pmu 1v0m 2ay2 2gz8 2ooh 2va7 2xyr 3bxg 3fal 3ipb 3mzc 3qlb 3tmk 3zrl 4ce3 4fxz 4j8b 4mdn 4pn1 1a9m 1fq8 1kz8 1pmv 1v0n 2ay3 2gzl 2ooz 2vaq 2xys 3bxh 3fas 3ipe 3n0h 3qlm 3tn8 3zrm 4ceb 4fyh 4j8g 4mdq 4pni 1a9q 1fsg 1kzk 1pmx 1v0o 2ay4 2h02 2op3 2vb8 2xyt 3bxs 3fat 3iph 3n0n 3qmk 3tne 3zs0 4cfe 4fys 4j8m 4mdr 4pnk 1a9u 1fsw 1kzn 1pop 1v0p 2ay5 2h03 2op9 2vba 2xyu 3bxz 3fbr 3ipq 3n1c 3qn7 3tnh 3zs1 4cff 4fz3 4j8s 4mds 4pnl 1aaq 1fsy 1l0a 1pot 1v11 2ay6 2h13 2opb 2vc7 2xz5 3bym 3fc1 3ips 3n1v 3qnd 3tpp 3zso 4cfl 4fz6 4j8t 4mdt 4pnm 1abf 1ft4 1l2s 1ppc 1v16 2ay7 2h15 2oph 2vc9 2xzg 3byo 3fc2 3ipu 3n1w 3qnj 3tpr 3zsq 4cfm 4fzc 4j93 4men 4pnn 1abt 1fta 1l2z 1pph 1v1j 2ay8 2h1h 2opy 2vcb 2xzq 3bys 3fc8 3ipx 3n23 3qo2 3tpu 3zst 4cfu 4fzg 4j9a 4meo 4pnq 1acj 1ftj 1l5q 1ppi 1v1k 2ay9 2h21 2oq6 2vcg 2y06 3byu 3fcb 3ipy 3n2c 3qo3 3tpx 3zsw 4cfv 4fzj 4jai 4mep 4pnr 1ad8 1ftk 1l5r 1ppl 1v1m 2ayp 2h23 2oqi 2vci 2y07 3bz3 3fcf 3iqg 3n2e 3qo9 3ts4 3zsx 4cfw 4g0a 4jaj 4meq 4pns 1add 1ftl 1l5s 1ppm 1v2h 2ayr 2h2d 2oqs 2vcj 2y0j 3bze 3fci 3iqh 3n2p 3qox 3tsd 3zsy 4cfx 4g0c 4jal 4mex 4pnt 1adl 1ftm 1l6m 1ppw 1v2j 2az5 2h2e 2oqv 2vcw 2y1d 3bzf 3fck 3iqi 3n2u 3qoz 3tsk 3zsz 4cg8 4g0k 4jaz 4mf0 4po0 1ado 1fv0 1l6s 1ppx 1v2k 2az8 2h2g 2or4 2vcx 2y1g 3bzi 3fcl 3iqj 3n2v 3qpn 3tsz 3zt1 4cg9 4g0l 4jbl 4mf1 4po7 1af2 1fv9 1l6y 1pq3 1v2l 2az9 2h2h 2or9 2vd0 2y1n 3c0z 3fcq 3iqq 3n35 3qpo 3tt0 3zt2 4cga 4g0p 4jbo 4mfe 4poh 1af6 1fvt 1l7x 1pq6 1v2m 2azb 2h3e 2ork 2vd1 2y1o 3c10 3fdn 3iqu 3n3g 3qpp 3tt4 3zt4 4cgi 4g0q 4jbp 4mg5 4poj 1afk 1fvv 1l83 1pqc 1v2n 2azc 2h42 2os9 2vd4 2y1w 3c14 3fdt 3iqv 3n3j 3qps 3tti 3ztc 4cgj 4g0y 4jbs 4mg6 4pow 1afl 1fw0 1l8g 1pr1 1v2o 2azm 2h44 2osc 2vd7 2y1x 3c1k 3fe7 3irx 3n3l 3qqa

3ttj 3ztd 4ch2 4g0z 4jc1 4mg7 4pox 1ag9 1fwe 1la3 1pr5 1v2p 2azr 2h4g 2osf 2ves 2y2h 3c1n 3fea 3is9 3n45 3qqk 3ttm 3ztx 4ch8 4g11 4jck 4mg8 4pp0 1agm 1fwu 1laf 1prl 1v2q 2b07 2h4k 2osm 2veu 2y2i 3c1x 3fed 3isj 3n46 3qqs 3ttn 3zuk 4ci1 4g16 4jda 4mg9 4pp3 1aht 1fwv 1lag 1prm 1v2r 2b17 2h4n 2ot1 2vev 2y2j 3c2f 3fee 3iss 3n49 3qqu 3ttp 3zv7 4ci2 4g17 4jdf 4mga 4pp5 1ahx 1fyr 1lah 1pro 1v2s 2b1g 2h5a 2ou7 2vew 2y2k 3c2o 3feg 3isw 3n4c 3qri 3ttz 3zvt 4ci3 4g19 4je7 4mgb 4pp7 1ahy 1fzj 1lan 1ps3 1v2t 2b1i 2h5d 2ov4 2vex 2y2n 3c2r 3fei 3itc 3n4l 3qrj 3tu1 3zvw 4cig 4g1f 4je8 4mgc 4pp9 1ai4 1fzk 1lb6 1pu8 1v2u 2b1p 2h5e 2ovq 2vey 2y2p 3c2u 3fej 3ith 3n51 3qrk 3tu7 3zvy 4cik 4g2f 4jfd 4mgd 4ppa 1ai5 1fzm 1lbf 1pum 1v2v 2b1q 2h5i 2ovv 2vf6 2y34 3c39 3ff3 3itu 3n5e 3qs1 3tu9 3zw3 4civ 4g2j 4jfe 4mgv 4ppb 1ai6 1fzo 1lbk 1pun 1v2w 2b1r 2h5j 2ovx 2vfk 2y36 3c3o 3ffg 3itz 3n5h 3qs4 3tun 3zxe 4ciw 4g2l 4jff 4mh7 4ppc 1ai7 1fzq 1lcj 1puq 1v3x 2b1v 2h65 2ovy 2vfz 2y3p 3c3q 3ffp 3iu7 3n5j 3qs5 3tv4 3zxh 4cix 4g2r 4jfi 4mha 4pqn 1aid 1g05 1lcp 1pus 1v41 2b1z 2h6b 2ovz 2vgc 2y4a 3c3r 3ffu 3iu8 3n5k 3qs6 3tv5 3zxv 4ciy 4g2w 4jfj 4mho 4pr5 1aj6 1g1d 1ld7 1pvn 1v48 2b2v 2h6k 2ow0 2vgo 2y4k 3c45 3fgc 3iu9 3n5u 3qs8 3tv6 3zxz 4ciz 4g2y 4jfk 4mhs 4pra 1aj7 1g1e 1ld8 1pw6 1v79 2b4l 2h6q 2ow2 2vgp 2y4l 3c4c 3fh5 3iub 3n6k 3qsb 3tv7 3zy2 4cj4 4g31 4jfl 4mhy 4prb 1ajn 1g27 1lee 1pwp 1v7a 2b4m 2h6t 2ow3 2vh0 2y4m 3c4e 3fh7 3iuc 3n75 3qsd 3tv8 3zy5 4cjp 4g34 4jfm 4mhz 4prd 1ajp 1g2a 1leg 1pwq 1vcj 2b52 2h8h 2ow6 2vh6 2y4s 3c4f 3fhb 3iue 3n76 3qt6 3tvc 3zya 4cjq 4g3f 4jfs 4mi3 4pre 1ajq 1g2k 1lek 1pwu 1vcu 2b53 2h96 2ow7 2vhj 2y54 3c4h 3fhe 3iut 3n7a 3qt7 3tvl 3zyb 4cjr 4g3g 4jft 4mi6 4prg 1ajv 1g2l 1lev 1pwy 1vea 2b54 2h9m 2ow9 2vhq 2y56 3c52 3fhr 3iux 3n7h 3qtf 3tvw 3zyf 4ck3 4g4p 4jfv 4mi9 4prh 1ajx 1g2m 1lf2 1px4 1veb 2b55 2h9n 2owb 2vi5 2y57 3c56 3fhs 3ivc 3n7o 3qti 3tvx 3zyh 4cki 4g55 4jfw 4mib 4pri 1akq 1g2o 1lf3 1pxh 1vgc 2b5j 2h9p 2oxd 2vie 2y58 3c5u 3fi2 3ivg 3n7r 3qto 3twd 3zyr 4ckj 4g5f 4jfx 4mic 4prj 1akr 1g30 1lf8 1pxi 1vij 2b7a 2h9t 2oxn 2vin 2y59 3c6t 3fi3 3ivh 3n7s 3qtq 3twj 3zyu 4ckr 4g5y 4jfz 4mji 4prn 1akt 1g32 1lf9 1pxj 1vik 2b7d 2ha0 2oxx 2vio 2y5f 3c6u 3fj7 3ivi 3n86 3qtr 3twp 3zze 4cku 4g68 4jg0 4mjo 4prp 1aku 1g35 1lfo 1pxk 1vj5 2b7f 2ha2 2oxy 2vip 2y5g 3c6w 3fjg 3ivq 3n8k 3qts 3twr 3zzf 4cl9 4g8l 4jg1 4mjp 4ps3 1akv 1g36 1lgt 1pxl 1vj6 2b8l 2ha4 2oyk 2viq 2y5h 3c72 3fjz 3ivv 3n8n 3qtu 3tws 3zzh 4clb 4g8m 4jg6 4mjq 4ps5 1akw 1g37 1lgw 1pxm 1vj9 2b8v 2ha5 2oyl 2viv 2y5k 3c79 3fk1 3ivx 3n9r 3qtv 3twu 456c 4cli 4g8n 4jg7 4mjr 4ps8 1al7 1g3b 1lhc 1pxn 1vja 2b9a 2ha6 2oym 2viw 2y5l 3c7n 3fkt 3iw4 3n9s 3qtw 3twv 4a0j 4clj 4g8o 4jg8 4mk0 4psb 1al8 1g3c 1lhd 1pxo 1vjb 2baj 2ha7 2oz2 2viy 2y67 3c7p 3fkv 3iw5 3nal 3qtx 3tww 4a16 4clp 4g8r 4jgv 4mk1 4psh 1alw 1g3d 1lhe 1pxp 1vjc 2bak 2hah 2oz5 2viz 2y68 3c7q 3fl5 3iw6 3nam 3qtz 3twx 4a1u 4clz 4g8v 4jh0 4mk2 4psq 1amk 1g3e 1lhf 1py1 1vjd 2bal 2hai 2oz6 2vj1 2y6c 3c84 3fl8 3iw7 3nan 3qu0 3txo 4a1w 4cmo 4g8y 4jhq 4mk5 4psx 1amn 1g3f 1lhg 1py2 1vjy 2ban 2haw 2oz7 2vj6 2y6d 3c88 3fl9 3iw8 3nb5 3que 3ty0 4a22 4cmt 4g90 4jht 4mk7 4puj 1amw 1g3m 1lhu 1py5 1vkj 2bb7 2hb1 2ozr 2vj7 2y6o 3c89 3fmq 3iww 3nba 3qup 3tyq 4a23 4cmu 4g93 4jhz 4mk8 4puk 1anf 1g42 1lhv 1pye 1vot 2bba 2hb3 2p09 2vj8 2y6s 3c8a 3fmr 3iwy 3nc4 3qvu 3tyv 4a2a 4cnh 4g95 4jia 4mk9 4pul 1ao0 1g45 1lhw 1pyg 1vr1 2bbb 2hb9 2p0d 2vj9 2y71 3c8b 3fmz 3ixg 3nc9 3qvv 3tz0 4a4c 4cp7 4g9c 4jib 4mka 4pum 1ao8 1g46 1li2 1pyn 1vrt 2bcd 2hd1 2p0x 2vjx 2y76 3c8e 3fn0 3ixj 3ncg 3qw5 3tz2 4a4e 4cpq 4ga3 4jik 4mlu 4puz 1apb 1g48 1li3 1pyw 1vru 2bdf 2hd6 2p15 2vk2 2y77 3c94 3fnm 3ixk 3ncq 3qw6 3tz4 4a4f 4cpr 4gah 4jin 4mm4 4pv0 1apv 1g49 1ljt 1pz5 1vsn 2bdj 2hdr 2p16 2vk6 2y7i 3c9e 3fnu 3jdw 3ncr 3qw7 3tza 4a4g 4cps 4gao 4jit 4mm5 4pvo 1apw 1g4j 1lke 1pzi 1vwf 2bdl 2hds 2p2a 2vkm 2y7k 3caj 3fpd 3jpv 3ncz 3qw8 3tzd 4a4h 4cpt 4gb9 4jj7 4mm6 4pvt 1aq1 1g4k 1lkk 1pzj 1vwl 2bdy 2hdu 2p2h 2vl1 2y7w 3cbp 3fpm 3jpx 3ndm 3qwc 3tzm 4a4l 4cpu 4gbd 4jj8 4mm7 4pvv 1aq7 1g4o 1lkl 1pzo 1vwn 2be2 2hdx 2p2i 2vl4 2y7x 3cbs 3fq7 3jq7 3nee 3qx5 3u0d 4a4o 4cpw 4gbz 4jje 4mm8 4px6 1aqc 1g50 1lkx 1pzp 1vyf 2bed 2hf8 2p33 2vl8 2y80 3ccb 3fqa 3jq8 3nef 3qx8 3u0p 4a4q 4cpx 4gcj 4jjf 4mm9 4pxf 1aqi 1g52 1ll4 1q0b 1vyg 2bes 2hfp 2p3a 2vle 2y81 3ccc 3fqe 3jq9 3neg 3qx9 3u0t 4a4v 4cpy 4gd6 4jjg 4mma 4py1 1aqj 1g53 1llb 1q1g 1vyj 2bet 2hh5 2p3b 2vmc 2y82 3ccn 3fqh 3jqa 3neo 3qxc 3u10 4a4w 4cpz 4gdy 4jjm 4mme 4pyn 1at5 1g54 1lnm 1q1m 1vyq 2bfq 2hha 2p3c 2vmd 2y8c 3cct 3fqk 3jqb 3nes 3qxd 3u15 4a4x 4cqe 4ge1 4jjq 4mmf 4pyo 1at6 1g5f 1lol 1q1y 1vyw 2bfr 2hhn 2p3d 2vmf 2y8i 3ccw 3fqs 3jqf 3new 3qxh 3u18 4a50 4cqf 4ge2 4jjs 4mmm 4pyq 1atl 1g5s 1loq 1q3d 1vyz 2bgd 2hiw 2p3g 2vnf 2y8l 3ccz 3fr2

3jqg 3nex 3qxm 3u1i 4a51 4cqg 4ge4 4jju 4mmp 4pyv 1atr 1g6g 1lor 1q3w 1vzq 2bge 2hiz 2p3i 2vnm 2y8o 3cd0 3fr4 3jrs 3nf3 3qxp 3u1y 4a6b 4cs9 4ge5 4jk6 4mn3 4pz5 1au0 1g6r 1los 1q41 1w0y 2bgn 2hj4 2p3o 2vnn 2y8q 3cd5 3fr5 3jrx 3nf6 3qxt 3u2k 4a6c 4csd 4ge6 4jkt 4mnp 4pz8 1au2 1g6s 1lox 1q4k 1w0z 2bgr 2hjb 2p4i 2vnp 2y9g 3cd7 3frg 3jsi 3nf7 3qxv 3u2q 4a6l 4csj 4ge7 4jkw 4mnq 4pzv 1auj 1g7f 1lpg 1q4l 1w10 2bj4 2hk5 2p4j 2vnt 2y9q 3cd8 3frz 3jsw 3nf8 3qyy 3u3f 4a6s 4ctj 4ge9 4jlh 4mnv 4pzw 1avd 1g7g 1lpk 1q4w 1w11 2bjm 2hkf 2p4s 2vo4 2ya6 3cda 3fsj 3juk 3nf9 3qzq 3u3u 4a6v 4ctk 4gee 4jlj 4mnw 4pzx 1avn 1g7p 1lpz 1q4x 1w12 2bks 2hl4 2p4y 2vo5 2ya7 3cdb 3fsm 3juo 3nfk 3qzt 3u3z 4a6w 4cts 4gfd 4jlm 4mnx 4q0a 1avp 1g7q 1lq2 1q54 1w13 2bkt 2hm1 2p53 2vo7 2ya8 3cde 3ft2 3jup 3nfl 3qzv 3u4h 4a7b 4cu1 4gfm 4jln 4mny 4q0k 1aw1 1g7v 1lqd 1q5k 1w14 2bkz 2hmb 2p59 2vot 2yac 3ce0 3ft3 3juq 3ng4 3r00 3u4i 4a7c 4cu7 4gfn 4jls 4mo4 4q18 1awf 1g85 1lqe 1q5l 1w1d 2bmc 2hmh 2p7a 2vpe 2yay 3ce3 3ft4 3jvk 3nga 3r01 3u4o 4a7i 4cu8 4gfo 4jmg 4mo8 4q19 1awh 1g98 1lqf 1q63 1w1g 2bmg 2hmu 2p7g 2vpg 2yaz 3cen 3ft5 3jvr 3nhi 3r02 3u4r 4a7j 4cwf 4gg5 4jmh 4mot 4q1c 1awi 1g9a 1lrh 1q65 1w1p 2bmk 2hmv 2p7z 2vpn 2yb0 3cf1 3ft8 3jvs 3nht 3r04 3u4u 4a95 4cwn 4gg7 4jmu 4mp2 4q1e 1ax0 1g9b 1lrt 1q66 1w1t 2bmv 2hmw 2p83 2vpo 2yb9 3cf8 3ftq 3jwq 3nif 3r0h 3u4w 4a9c 4cwo 4ggl 4jmx 4mp7 4q1f 1ax1 1g9c 1lst 1q6j 1w1v 2bmz 2hnc 2p8h 2vpp 2ybk 3cf9 3fts 3jwr 3nii 3r0i 3u51 4a9i 4cwp 4ggz 4jn2 4mpc 4q1n 1ax2 1g9d 1lt5 1q6k 1w1y 2bo4 2hnx 2p8n 2vqj 2ybp 3cfn 3ftu 3jxw 3nij 3r0t 3u5j 4a9m 4cwq 4gh6 4jn4 4mpe 4q3t 1axr 1g9r 1lt6 1q6m 1w22 2boh 2hny 2p8o 2vqm 2ybs 3cfs 3ftv 3jy0 3nik 3r0w 3u5l 4a9n 4cwr 4ghi 4jne 4mpn 4q3u 1axs 1g9s 1lv8 1q6n 1w25 2boi 2hob 2p8s 2vqt 2ybt 3cft 3ftw 3jy9 3nil 3r0y 3u6a 4a9r 4cws 4ght 4jnj 4mq1 4q4o 1axz 1g9t 1lvc 1q6p 1w2g 2boj 2hoc 2p93 2vr0 2ybu 3cfv 3fty 3jya 3nim 3r16 3u6h 4a9s 4cwt 4gid 4jnm 4mq2 4q4p 1ayu 1ga8 1lvk 1q6s 1w2h 2bok 2hog 2p94 2vr3 2yc3 3cgf 3ftz 3jyj 3nin 3r1v 3u6i 4a9t 4cxw 4gih 4joa 4mqp 4q4q 1ayv 1ga9 1lvu 1q6t 1w2k 2bow 2hpa 2p95 2vr4 2yc5 3cgo 3fu0 3jyr 3njq 3r21 3u6j 4a9u 4cxx 4gii 4joe 4mqu 4q4r 1aze 1gaf 1lxf 1q72 1w2x 2bpv 2hqu 2p98 2vrj 2ycf 3cgy 3fu3 3jzc 3njy 3r22 3u6w 4aa0 4cxy 4giu 4jof 4mr3 4q4s 1azg 1gag 1lxh 1q7a 1w31 2bpx 2hr6 2p99 2vrx 2ych 3ch9 3fu5 3jzf 3nk8 3r24 3u78 4aa1 4cy1 4gj2 4jog 4mr4 4q6d 1azl 1gah 1lyb 1q83 1w3j 2bq6 2hrm 2p9a 2vsl 2ycm 3chc 3fu6 3jzg 3nkk 3r2a 3u7k 4aa2 4d08 4gj3 4joh 4mr5 4q6e 1azm 1gai 1lyx 1q84 1w3k 2bq7 2hrp 2pax 2vt3 2ycq 3chd 3fuc 3jzi 3nkx 3r2b 3u7l 4aa4 4d09 4gj6 4joj 4mr6 4q6r 1azx 1gar 1lzo 1q8t 1w3l 2bqv 2hs1 2pbw 2vta 2ycr 3che 3fud 3jzj 3nlb 3r2f 3u7m 4aa5 4d0w 4gj7 4jok 4mra 4q93 1b05 1gbq 1lzq 1q8u 1w4l 2bqw 2hs2 2pcp 2vtd 2ycs 3chf 3fue 3jzk 3nm6 3r2y 3u7n 4aa7 4d0x 4gj8 4joo 4mrd 4q9m 1b0f 1gbt 1m0b 1q8w 1w4o 2br1 2hu6 2pcu 2vte 2yde 3chg 3fuf 3jzo 3nmq 3r42 3u7s 4ab8 4d1a 4gj9 4jp9 4mre 4q9o 1b0h 1gca 1m0n 1q91 1w4p 2br8 2hug 2pe0 2vth 2ydf 3cho 3fuh 3jzp 3nnu 3r4m 3u81 4ab9 4d1b 4gja 4jpa 4mrf 4q9s 1b11 1gcz 1m0o 1q95 1w4q 2brb 2hvx 2pe1 2vti 2ydi 3chp 3fui 3jzq 3nnv 3r4n 3u8d 4aba 4d1c 4gjb 4jpc 4mrg 4q9z 1b1h 1gfw 1m0q 1q9d 1w51 2brc 2hw2 2pe2 2vtj 2ydj 3chq 3fuj 3jzr 3nnw 3r4o 3u8h 4abb 4d1d 4gjc 4jpe 4mrh 4qaa 1b2h 1gfy 1m13 1q9m 1w5v 2brg 2hwg 2peh 2vtl 2ydk 3chr 3fuk 3jzs 3nnx 3r4p 3u8j 4abd 4d1j 4gjd 4jps 4mro 4qab 1b2i 1gfz 1m1b 1qan 1w5w 2brh 2hwh 2pem 2vtm 2ydm 3chs 3ful 3k00 3nok 3r5j 3u8k 4abe 4d1s 4gk2 4jpx 4mrw 4qac 1b2m 1ggd 1m21 1qaq 1w5x 2brm 2hwi 2pfy 2vtn 2ydo 3cib 3fum 3k02 3nox 3r5m 3u8l 4abf 4d1y 4gk3 4jpy 4mrz 4qag 1b32 1ggn 1m2p 1qaw 1w5y 2brn 2hwo 2pg2 2vto 2ydt 3cic 3fun 3k05 3np7 3r5n 3u8m 4abg 4d2d 4gk4 4jq7 4ms0 4qdk 1b38 1ghv 1m2q 1qb1 1w6h 2bro 2hwp 2pgj 2vtp 2ydv 3cii 3fup 3k0k 3np9 3r5t 3u8n 4abh 4d2p 4gk7 4jq8 4msa 4qem 1b39 1ghw 1m2r 1qb6 1w6j 2brp 2hxl 2pgz 2vtq 2ydw 3cj2 3fur 3k15 3npc 3r69 3u8w 4abi 4d2t 4gkc 4jql 4msc 4qer 1b3f 1ghy 1m2x 1qb9 1w6r 2bt9 2hxq 2ph6 2vtr 2ye9 3cj3 3fuz 3k16 3nq3 3r6c 3u90 4abj 4d2v 4gkh 4jr0 4mse 4qf7 1b3g 1ghz 1m48 1qbn 1w6y 2btr 2hy0 2ph8 2vts 2yek 3cj4 3fv1 3k1j 3nq9 3r6g 3u92 4abk 4d2w 4gki 4jr3 4msg 4qf8 1b3h 1gi1 1m4h 1qbo 1w70 2bts 2hyy 2ph9 2vtt 2yel 3cj5 3fv2 3k22 3nrm 3r6t 3u93 4abu 4d3h 4gkm 4jr5 4msk 4qfg 1b3l 1gi4 1m51 1qbq 1w76 2bu5 2hz0 2phb 2vu3 2yem 3cjf 3fv3 3k26 3nrz 3r6u 3u9c 4abv 4d83 4glr 4jrg 4msl 4qfr 1b40 1gi6 1m5b 1qbr 1w7g 2bua 2hz4 2pix 2vuk 2yer 3cjo 3fv7 3k27 3ns7 3r7b 3u9n 4ac3 4d85 4glx 4jrv 4msn 4qfs 1b42 1gi7 1m5c 1qbs 1w7h 2bub 2hzi 2piy 2vur 2yex 3ck7 3fv8 3k2f 3ns9 3r7n 3u9q 4acc 4d88 4gly 4jsa 4mss 4qg7 1b46 1gi8 1m5d 1qbu 1w7x 2buc 2hzl 2piz 2vv9 2yfa 3ck8 3fvg 3k37 3nsh 3r7o 3ua8

4acd 4d89 4gm3 4jsc 4msu 4qga 1b4d 1gi9 1m5e 1qbv 1w80 2buv 2hzn 2pj0 2vvc 2yfe 3ckb 3fvh 3k39 3nsn 3r7q 3ua9 4acf 4d8a 4gm8 4jsr 4mt9 4qgd 1b4h 1gj4 1m5f 1qca 1w82 2bv4 2hzy 2pj1 2vvo 2yg2 3cke 3fvl 3k3a 3nsq 3r7r 3ual 4acg 4d8c 4gmc 4jss 4mti 4qge 1b4z 1gj5 1m5w 1qcp 1w83 2bvd 2i03 2pj2 2vvs 2yga 3ckp 3fvn 3k3b 3nth 3r88 3uat 4ach 4d8e 4gmy 4jsz 4mu7 4qgf 1b51 1gj6 1m6p 1qf0 1w84 2bve 2i0a 2pj3 2vvt 2yge 3ckr 3fw3 3k3e 3nti 3r8i 3ubd 4aci 4d8i 4gne 4jt8 4muv 4qgg 1b52 1gj7 1m74 1qf1 1w8l 2bvr 2i0d 2pj4 2vvu 2ygf 3ckt 3fw4 3k3g 3ntp 3r8u 3ubx 4acm 4d8n 4gnf 4jt9 4muw 4qgh 1b55 1gj8 1m7d 1qf2 1w8m 2bvs 2i0e 2pj5 2vvv 2ygu 3ckz 3fwv 3k3h 3nu3 3r8v 3ucj 4acu 4d8s 4gng 4ju3 4mvh 4qgi 1b56 1gja 1m7i 1qf4 1w96 2bvx 2i0g 2pj6 2vw1 2yhd 3cl0 3fx6 3k3i 3nu4 3r8z 3ud5 4acx 4d8z 4gny 4ju4 4mvw 4qh7 1b57 1gjb 1m7q 1qf5 1w9u 2bw7 2i0h 2pj7 2vw2 2yhw 3clp 3fxb 3k3j 3nu5 3r91 3ud7 4ad2 4d9p 4gpl 4ju6 4mvx 4qh8 1b58 1gjd 1m7y 1qfs 1w9v 2bxt 2i0j 2pj8 2vw5 2yhy 3cm2 3fxv 3k41 3nu6 3r92 3ud8 4ad3 4da5 4gq4 4ju7 4mvy 4qhp 1b5g 1gmy 1m9n 1qft 1waw 2bxu 2i0v 2pj9 2vwc 2yi0 3cm7 3fxw 3k48 3nu9 3r93 3ud9 4ad6 4daf 4gq6 4jv6 4mw0 4qij 1b5h 1gni 1mai 1qhc 1wax 2byh 2i0y 2pja 2vwf 2yi5 3cn0 3fxz 3k4d 3nuj 3r9d 3uda 4ael 4dai 4gql 4jv7 4mw1 4qir 1b5i 1gnj 1mau 1qhr 1way 2byi 2i19 2pjb 2vwl 2yi7 3co9 3fy0 3k4q 3nuo 3r9h 3udd 4af3 4daw 4gqp 4jv8 4mw2 4qjr 1b5j 1gnm 1maw 1qi0 1wb0 2byr 2i1m 2pjc 2vwm 2yig 3coh 3fyj 3k5c 3nus 3r9n 3udh 4afe 4db7 4gqq 4jv9 4mw4 4qk4 1b6j 1gnn 1mcz 1qin 1wbg 2bys 2i1r 2pjl 2vwn 2yim 3coj 3fyk 3k5d 3nuu 3r9o 3udj 4afg 4dbm 4gqr 4jvb 4mw5 4qkd 1b6k 1gno 1mdl 1qiw 1wbn 2bz5 2i2b 2pjt 2vwo 2yiq 3cow 3fyz 3k5f 3nuy 3rah 3udk 4afh 4dcd 4gr3 4jve 4mw6 4qlq 1b6l 1gny 1me3 1qj1 1wbo 2bz6 2i2c 2pk5 2vwu 2yir 3coy 3fz1 3k5g 3nw3 3rak 3udm 4afj 4dce 4gr8 4jvi 4mw7 4qls 1b6m 1gpk 1me4 1qj6 1wbs 2bz8 2i3h 2pk6 2vwv 2yis 3coz 3fzc 3k5i 3nw5 3ral 3udn 4aft 4dcs 4gs6 4jvj 4mw9 4qlt 1b6p 1gpn 1me7 1qj7 1wbt 2bza 2i3i 2pks 2vww 2yit 3cp9 3fzn 3k5k 3nw6 3rbm 3udp 4ag8 4dcv 4gs8 4jvq 4mwb 4qlu 1b74 1gpy 1me8 1qja 1wbv 2bzz 2i3v 2pl0 2vwx 2yiu 3cpb 3fzr 3k5u 3nw7 3rbq 3udq 4agc 4dcx 4gs9 4jwk 4mwc 4qlv 1b7h 1gqs 1mem 1qjb 1wbw 2c02 2i3z 2pl9 2vwy 2yiv 3cpc 3fzs 3k5v 3nw9 3rbu 3udr 4agd 4dcy 4gsc 4jwr 4mwe 4qme 1b8n 1grp 1mes 1qji 1wc1 2c0o 2i40 2pll 2vwz 2yiw 3cph 3fzt 3k5x 3nwb 3rcd 3udv 4agl 4ddh 4gsy 4jx7 4mwq 4qn7 1b8o 1gsf 1met 1qk3 1wc6 2c1a 2i47 2pmc 2vx0 2yix 3cpj 3fzy 3k7f 3nwe 3rcj 3udy 4agm 4ddk 4gtm 4jx9 4mwr 4qnb 1b8y 1gsz 1meu 1qk4 1wcc 2c1b 2i4d 2pmk 2vx1 2yj2 3cqu 3g08 3k83 3nww 3rde 3ue4 4agn 4ddl 4gto 4jxs 4mwu 4qo4 1b9j 1gt1 1mf4 1qka 1wcq 2c1n 2i4j 2pmn 2vx9 2yj8 3cqw 3g0b 3k84 3nx7 3rdh 3uec 4ago 4ddm 4gtp 4jxv 4mwv 4qok 1b9s 1gt3 1mfa 1qkb 1wdn 2c1p 2i4p 2pnc 2vxa 2yj9 3cr4 3g0c 3k8c 3nxq 3rdo 3ued 4agp 4dds 4gtq 4jxw 4mww 4qpl 1b9t 1gt4 1mfd 1qkn 1wdq 2c1q 2i4t 2pnx 2vxn 2yjb 3cr5 3g0d 3k8d 3ny3 3rdq 3uef 4agq 4ddy 4gtr 4jyb 4mwx 4qrc 1b9v 1gt5 1mfg 1qkt 1wdr 2c2l 2i4u 2pog 2vyt 2yjc 3cs7 3g0e 3k8o 3nyd 3rdv 3ueo 4ah9 4de0 4gts 4jyc 4mwy 4qsh 1ba8 1gu1 1mfi 1qku 1wdy 2c3i 2i4v 2poq 2vzr 2yjq 3cs8 3g0f 3k8q 3nyn 3re4 3ueu 4ahr 4de1 4gtt 4jym 4mx0 4qsk 1bai 1gu3 1mh5 1ql7 1we2 2c3j 2i4w 2pou 2w05 2yjw 3csl 3g0g 3k97 3nyx 3rey 3uev 4ahs 4de2 4gtv 4jyt 4mx1 4qsm 1bap 1gui 1mhw 1ql9 1wht 2c3k 2i4x 2pov 2w06 2yjx 3cso 3g0i 3k98 3nzc 3rf4 3uew 4ahu 4de3 4gu6 4jyu 4mx5 4qsu 1bb0 1guw 1mik 1qm4 1wkm 2c3l 2i4z 2pow 2w08 2yk1 3cst 3g0w 3k99 3nzi 3rf5 3uex 4ahv 4de5 4gu9 4jyv 4mx9 4qsv 1bbz 1gux 1mj7 1qm5 1wn6 2c4f 2i5f 2pq9 2w0d 2yk9 3cth 3g15 3k9x 3nzk 3rhk 3uf9 4ai5 4de7 4gue 4jz1 4myd 4qsw 1bcd 1gvw 1mjj 1qng 1wok 2c4g 2i6a 2pqb 2w0j 2ykb 3ctj 3g19 3ka2 3nzs 3rhx 3ufa 4ai8 4dea 4gui 4jzb 4myh 4qsx 1bcj 1gvx 1mkd 1qnh 1wqv 2c4v 2i6b 2pqc 2w0p 2ykc 3ctq 3g1d 3kab 3nzu 3ri1 3ufl 4aia 4deb 4guj 4jzd 4myq 4qt0 1bcu 1gwm 1ml1 1qon 1ws1 2c57 2i72 2pqj 2w0s 2yke 3ctr 3g1m 3kac 3nzw 3rik 3ug2 4aif 4ded 4gv1 4jze 4mz4 4qta 1bdl 1gwq 1mm6 1qpb 1ws4 2c5n 2i7c 2pql 2w0x 2yki 3ctt 3g1v 3kad 3nzx 3ril 3ugc 4aig 4deg 4gv8 4jzf 4mz5 4qtb 1bdq 1gwr 1mm7 1qpe 1ws5 2c5o 2i80 2pqz 2w0z 2ykj 3cvk 3g2h 3kaf 3o0e 3rin 3uh2 4aj2 4deh 4gvm 4jzi 4mz6 4qtc 1bdr 1gwv 1mmp 1qq9 1wss 2c5x 2ica 2pr9 2w10 2ylc 3cwe 3g2i 3kag 3o0g 3rj7 3uh4 4aj4 4del 4gvu 4jzr 4mzf 4qtd 1bgo 1gww 1mmq 1qr3 1wtg 2c5y 2idk 2pre 2w12 2yln 3cwj 3g2j 3kah 3o0j 3rjc 3uhm 4aje 4dem 4gw1 4k0o 4mzh 4qtn 1bgq 1gx0 1mmr 1qs4 1wu1 2c69 2idw 2pri 2w16 2ylo 3cwk 3g2k 3kai 3o0u 3rjm 3ui2 4aji 4der 4gw5 4k0u 4mzj 4quo 1bhf 1gx4 1mn9 1qsc 1wug 2c6c 2ie4 2prj 2w17 2ylp 3cx9 3g2l 3kb3 3o1d 3rjw 3ui7 4ajk 4des 4gw6 4k0y 4mzk 4qxo 1bhx 1gx8 1mnc 1qti 1wum 2c6e 2ieh 2psj

2w1c 2ylq 3cy2 3g2n 3kb7 3o1e 3rk5 3uib 4ajl 4det 4gw8 4k10 4mzl 4qxq 1bil 1gym 1mns 1qtn 1wun 2c6g 2ien 2psu 2w1d 2ym3 3cyu 3g2s 3kba 3o1g 3rk7 3uig 4ajn 4deu 4gwi 4k18 4n00 4qxr 1bim 1gyx 1moq 1qvt 1wuq 2c6i 2ieo 2psv 2w1e 2ym5 3cyw 3g2t 3kbz 3o23 3rk9 3uih 4ajo 4dfb 4gwk 4k19 4n07 4qy3 1bio 1gyy 1mpa 1qvu 1wur 2c6k 2ig0 2psx 2w1f 2ym6 3cyx 3g2u 3kc0 3o2m 3rkb 3uii 4ajw 4dff 4gxl 4k2f 4n1b 4qye 1biw 1gz3 1mpl 1qw7 1wv7 2c6l 2igv 2pt9 2w1g 2ym7 3cyy 3g2v 3kc3 3o3j 3rkz 3uij 4akn 4dfg 4gxs 4k2g 4n1t 4qyg 1bji 1gz9 1mq1 1qwe 1wva 2c6m 2igw 2ptz 2w1h 2ym8 3cyz 3g2w 3kce 3o4k 3rl3 3uik 4al4 4dfl 4gy5 4k2y 4n1u 4qyh 1bjr 1gzc 1mq5 1qwf 1wvc 2c6n 2igx 2pu0 2w1i 2ymd 3cz1 3g2y 3kcf 3o4l 3rl4 3uil 4alg 4dfn 4gye 4k3h 4n3l 4qyy 1bju 1gzg 1mq6 1qx1 1wvj 2c6o 2igy 2pu1 2w26 2yme 3czv 3g2z 3kck 3o56 3rl7 3uix 4alu 4dfu 4gz3 4k3k 4n3r 4qzs 1bjv 1gzv 1mqd 1qxk 1wxz 2c80 2ihj 2pu2 2w2i 2ymt 3d04 3g30 3kd7 3o57 3rl8 3uj9 4alv 4dfw 4gzf 4k3l 4n3w 4r1e 1bkj 1h00 1mqg 1qxl 1wzy 2c8w 2ihq 2puy 2w2u 2ynd 3d0b 3g31 3kdb 3o5n 3rlb 3ujb 4alw 4dgb 4gzp 4k3m 4n4s 4r3w 1bkm 1h01 1mqh 1qxw 1x07 2c8x 2iit 2pv1 2w3i 2yne 3d0e 3g32 3kdc 3o5x 3rlp 3ujc 4alx 4dgg 4gzt 4k3n 4n4t 4r4c 1bky 1h07 1mqi 1qxy 1x0n 2c8y 2iiv 2pv2 2w3k 2ynn 3d14 3g34 3kdd 3o6l 3rlq 3ujd 4aml 4dgm 4gzw 4k3o 4n4v 4r4i 1bl4 1h08 1mqj 1qxz 1x11 2c90 2iko 2pv3 2w3l 2ynr 3d1e 3g35 3kdm 3o6m 3rlr 3ukr 4amw 4dgn 4gzx 4k3p 4n5d 4r4o 1bl6 1h0a 1mrn 1qy1 1x1z 2c92 2iku 2pvh 2w3o 2yns 3d1f 3g3d 3kdt 3o6t 3rm4 3uli 4amx 4dgo 4h1e 4k3q 4n5g 4r4q 1bl7 1h0r 1mrs 1qy2 1x38 2c93 2il2 2pvj 2w47 2yof 3d1g 3g3m 3kdu 3o75 3rm8 3umo 4amy 4dgr 4h1j 4k3r 4n5t 4r4t 1bm2 1h0w 1mrw 1qy5 1x39 2c94 2ilp 2pvk 2w4i 2yog 3d1v 3g3n 3kec 3o7u 3rm9 3ump 4amz 4dh6 4h1m 4k42 4n6g 4r59 1bm6 1h1b 1mrx 1qyg 1x6u 2c97 2imb 2pvl 2w4x 2yoh 3d1x 3g3r 3ked 3o84 3rme 3umq 4an0 4dhf 4h2j 4k43 4n6h 4r5b 1bm7 1h1d 1ms0 1r0p 1x70 2c9b 2imd 2pvm 2w54 2yoj 3d1y 3g42 3kej 3o86 3rmf 3umw 4an1 4dhl 4h2m 4k4e 4n6y 4r5g 1bma 1h1h 1ms6 1r0x 1x76 2c9d 2in6 2pvn 2w5i 2yol 3d1z 3g45 3kek 3o87 3rni 3umx 4an2 4dhm 4h2o 4k4f 4n6z 4r5n 1bmb 1h1p 1ms7 1r17 1x78 2c9t 2io6 2pvu 2w66 2ypi 3d20 3g4f 3ken 3o88 3ro0 3unj 4an3 4dhn 4h36 4k4j 4n70 4r5t 1bmk 1h1q 1msm 1r1h 1x7a 2ca8 2ioa 2pvv 2w67 2ypo 3d25 3g4g 3kf4 3o8g 3ro4 3unk 4an9 4dho 4h38 4k55 4n7e 4r5v 1bmm 1h1r 1msn 1r1i 1x7b 2cbj 2iog 2pvw 2w68 2ypp 3d27 3g4i 3kf7 3o8h 3roc 3unn 4anb 4dhp 4h39 4k5l 4n7g 4r5x 1bmn 1h1s 1mto 1r1j 1x7e 2cbs 2iok 2pwc 2w6c 2yq7 3d28 3g4k 3kfa 3o8p 3rpr 3unz 4anm 4dhq 4h3a 4k5m 4n7h 4r6t 1bmq 1h22 1mtr 1r2b 1x7q 2cbu 2ipo 2pwd 2w6m 2ywp 3d2e 3g4l 3kfc 3o95 3rpy 3uo4 4anq 4dhr 4h3b 4k5n 4n7j 4r6w 1bn1 1h23 1mu6 1r4w 1x7r 2cbv 2iqg 2pwg 2w6n 2yxj 3d2t 3g58 3kfn 3o96 3rq7 3uo5 4anu 4dhs 4h3c 4k5o 4n7m 4r6x 1bn3 1h24 1mu8 1r58 1x81 2cbz 2irz 2pwr 2w6o 2yz3 3d32 3g5d 3kga 3o99 3rqe 3uo6 4anv 4dht 4h3f 4k5p 4n7u 4r76 1bn4 1h25 1mue 1r5g 1x8b 2cc7 2is0 2py4 2w6p 2z1w 3d3p 3g5k 3kgp 3o9a 3rqf 3uo9 4anw 4dhu 4h3g 4k5y 4n7y 4r7m 1bnm 1h26 1mui 1r5h 1x8d 2ccb 2isc 2pyi 2w6q 2z3h 3d3x 3g5y 3kgq 3o9b 3rqg 3uod 4anx 4di2 4h3i 4k5z 4n84 4r8y 1bnn 1h28 1mv0 1r5n 1x8j 2ccc 2isv 2pym 2w6t 2z3z 3d45 3g6g 3kgt 3o9c 3rqw 3uoh 4aoc 4dij 4h3j 4k60 4n8d 4r91 1bnq 1h2k 1mwn 1r5v 1x8r 2ccs 2isw 2pyn 2w6u 2z4b 3d4f 3g6h 3kgu 3o9d 3rr4 3uoj 4aof 4dj7 4h3q 4k63 4n8e 4r92 1bnt 1h2t 1mx1 1r5w 1x8s 2cct 2it4 2pyy 2w6z 2z4o 3d4l 3g6m 3khj 3o9e 3rsb 3uok 4aoi 4djh 4h42 4k64 4n8q 4r93 1bnu 1h2u 1mxl 1r5y 1x8t 2ccu 2itk 2pze 2w70 2z4r 3d4q 3g6z 3khv 3o9f 3rse 3uol 4ap0 4djo 4h4b 4k66 4n8r 4r95 1bnv 1h35 1mxo 1r6g 1x9d 2ce9 2ito 2pzi 2w71 2z4w 3d4y 3g70 3kid 3o9g 3rsr 3up2 4ap7 4djp 4h4d 4k67 4n98 4rak 1bo5 1h36 1mxu 1r6n 1xa5 2cej 2itp 2pzy 2w73 2z4y 3d4z 3g72 3kig 3o9h 3rsv 3up7 4aph 4djq 4h4e 4k69 4n99 4rce 1bq3 1h37 1my2 1r6z 1xap 2cem 2itt 2q11 2w77 2z4z 3d50 3g76 3kiv 3o9i 3rsx 3upf 4apo 4djr 4h4m 4k6i 4n9a 4rcf 1bq4 1h39 1my3 1r78 1xb7 2cen 2ity 2q15 2w78 2z50 3d51 3g7l 3kjd 3o9l 3rt4 3uph 4app 4djs 4h58 4k6y 4n9b 4rdn 1bqm 1h3a 1my4 1r9l 1xbb 2ceo 2itz 2q1j 2w7x 2z52 3d52 3g86 3kjf 3o9p 3rt6 3upi 4apr 4dju 4h5c 4k6z 4n9c 4rfy 1bqn 1h3b 1my8 1rbo 1xbc 2ceq 2iu0 2q1l 2w7y 2z5o 3d5m 3g8e 3kjn 3o9v 3rt8 3upk 4aq3 4djv 4h5d 4k72 4n9d 4rfz 1bqo 1h3c 1mzc 1rbp 1xbo 2cer 2iuz 2q1q 2w85 2z5s 3d62 3g8i 3kjq 3oad 3rtf 3upv 4aq6 4djw 4h5e 4k75 4n9e 4rg0 1br5 1h3h 1mzs 1rd4 1xd0 2ces 2iv9 2q2a 2w87 2z5t 3d67 3g8o 3kku 3oaf 3rth 3upx 4aqc 4djx 4h71 4k76 4na4 4rio 1br6 1h46 1n0s 1rdi 1xdd 2cet 2ivu 2q2c 2w8f 2z60 3d6o 3g90 3kl6 3oag 3rti 3upy 4aqh 4djy 4h75 4k77 4na7 4rj3 1br8 1h4n 1n0t 1rdj 1xff 2cex 2iw4 2q2n 2w8g 2z6w 3d6p 3g9e 3kl8 3oap 3rtm 3upz 4ara 4dk5 4h7q 4k78 4na8 4rj4 1bsk 1h4w

1n1g 1rdl 1xfv 2cf8 2iw6 2q2y 2w8j 2z78 3d6q 3g9l 3km4 3oaw 3rtn 3uqf 4arb 4dk7 4h81 4k7i 4na9 4rj5 1bt6 1h5u 1n1m 1rdn 1xge 2cf9 2iw8 2q2z 2w8w 2z7h 3d78 3g9n 3kmc 3oay 3rtp 3uqg 4ark 4dk8 4h85 4k7n 4nah 4rj6 1btn 1h5v 1n1t 1rdt 1xgi 2cfd 2iw9 2q38 2w8y 2z7i 3d7b 3ga5 3kme 3ob0 3rtx 3uqp 4aro 4dko 4ha5 4k7o 4nak 4rj7 1bty 1h60 1n1v 1re1 1xgj 2cfg 2iws 2q3z 2w97 2z7r 3d7d 3gb2 3kmm 3ob1 3ru1 3uqr 4arw 4dkp 4hai 4k8o 4nal 4rj8 1bug 1h61 1n2v 1re8 1xh3 2cgf 2iwu 2q54 2w9h 2z8e 3d7f 3gba 3kmx 3ob2 3rul 3ur0 4as9 4dkq 4hbm 4k8s 4nan 4rlk 1bv7 1h62 1n3i 1rej 1xh4 2cgr 2iwx 2q55 2w9i 2z92 3d7g 3gbb 3kmy 3obq 3rum 3ur9 4asd 4dkr 4hbn 4k9h 4nat 4rll 1bv9 1h6e 1n3w 1rek 1xh5 2cgu 2iyf 2q5k 2w9r 2z94 3d7h 3gbe 3kn0 3obu 3run 3uri 4asj 4dkt 4hbp 4k9y 4nau 4rn6 1bwa 1h6h 1n3z 1rev 1xh6 2cgv 2izl 2q63 2wa4 2z97 3d7k 3gbq 3koo 3obx 3rup 3usn 4ask 4dld 4hbv 4kab 4nb3 4rpn 1bwb 1h79 1n43 1rgj 1xh7 2cgw 2izx 2q64 2wa8 2z9g 3d7m 3gc4 3kpu 3oc0 3rux 3usx 4asy 4dli 4hbw 4kai 4nb6 4rpo 1bwn 1h8l 1n46 1rgk 1xh9 2cgx 2j27 2q6b 2waj 2za0 3d7z 3gc5 3kpv 3ocb 3rv3 3ut5 4at3 4dma 4hbx 4kao 4nbk 4rqk 1bxl 1h8s 1n4h 1rgl 1xhm 2chm 2j2i 2q6f 2wap 2za3 3d83 3gc7 3kpw 3ocg 3rv4 3utu 4at4 4dmn 4hby 4kax 4nbl 4rrn 1bxo 1h8y 1n4k 1rhj 1xhy 2cht 2j2u 2q6h 2wb5 2za5 3d8w 3gcp 3kqa 3ocp 3rv6 3uu1 4at5 4dmw 4hco 4kb7 4nbn 4rro 1bxq 1h9l 1n4m 1rhk 1xjd 2chw 2j34 2q70 2wbb 2zas 3d8y 3gcq 3kqb 3oct 3rv7 3uug 4att 4dmy 4hct 4kb8 4ncn 4rrs 1bxr 1h9z 1n51 1rhm 1xk5 2chx 2j3q 2q72 2wbd 2zaz 3d8z 3gcu 3kqc 3ocz 3rv8 3uuo 4au7 4dn0 4hcu 4kb9 4ndu 4rrv 1byk 1ha2 1n5r 1rhq 1xk9 2chz 2j47 2q7m 2wbg 2zb0 3d91 3gcv 3kqd 3od0 3rv9 3uvk 4au8 4do3 4hcv 4kba 4ng9 4rsk 1bzc 1haa 1n5z 1rhr 1xka 2ci9 2j4a 2q7o 2wc3 2zb1 3d9k 3gds 3kqe 3odi 3rvg 3uvl 4aua 4do4 4hcz 4kbc 4nga 4sga 1bzf 1hbj 1n7i 1rhu 1xkk 2cia 2j4g 2q7q 2wc4 2zbk 3d9l 3ge7 3kqm 3odk 3rvi 3uvm 4auj 4do5 4hdb 4kbi 4ngh 4std 1bzh 1hbv 1n7j 1rin 1xlx 2cji 2j4i 2q7u 2wca 2zcq 3d9m 3gen 3kqo 3odl 3rw9 3uvn 4auy 4dow 4hdc 4kbk 4ngm 4tim 1bzj 1hc9 1n7m 1riw 1xlz 2ckm 2j4k 2q7y 2wcg 2zcr 3d9n 3gep 3kqp 3odu 3rwc 3uvo 4av0 4dpf 4hdf 4kby 4ngn 4tju 1bzs 1hdq 1n7t 1rjk 1xm4 2cle 2j4q 2q80 2wcx 2zcs 3d9o 3gev 3kqr 3oe0 3rwd 3uvp 4av4 4dpi 4hdp 4kcg 4ngp 4tjw 1bzy 1hdt 1n8u 1rlp 1xm6 2clf 2j4z 2q88 2wd1 2zda 3d9p 3gf2 3kqs 3oe4 3rwf 3uvq 4av5 4dpt 4he9 4kcx 4ngq 4tjy 1c12 1hee 1n8v 1rlq 1xmu 2cli 2j50 2q89 2wd3 2zdk 3d9v 3gfe 3kqt 3oe5 3rwg 3uvu 4avg 4dpu 4heg 4ke0 4ngr 4tk0 1c1r 1hef 1n94 1rm8 1xmy 2clk 2j62 2q8h 2wd7 2zdl 3d9z 3gfw 3kqw 3oe6 3rwh 3uvw 4avh 4dpy 4hej 4ke1 4ngs 4tk1 1c1u 1heg 1n95 1rmz 1xn0 2cll 2j6m 2q8i 2we3 2zdm 3da6 3ggc 3kqy 3oe8 3rwi 3uvx 4avi 4dq2 4heu 4keq 4ngt 4tk3 1c29 1hfs 1n9a 1rne 1xn2 2clm 2j75 2q8z 2web 2zdn 3da9 3ggj 3kr0 3oe9 3rwj 3uw4 4avj 4drk 4hev 4kfp 4nh7 4tk4 1c2t 1hge 1n9m 1rnm 1xn3 2clo 2j77 2q92 2wec 2zdt 3dab 3ggu 3kr1 3oeu 3rwp 3uw5 4avs 4drm 4hf4 4kg1 4nh8 4tk5 1c3b 1hgi 1nax 1rnt 1xnx 2clx 2j79 2q93 2wed 2zdx 3daj 3ggv 3kr2 3oev 3rx5 3uw9 4avt 4drn 4hfp 4kif 4nh9 4tkf 1c3e 1hgj 1nc1 1ro6 1xnz 2cm7 2j7b 2q94 2weg 2zdz 3daz 3ggw 3kr4 3of8 3rx7 3uwk 4avu 4dro 4hfz 4kij 4nhc 4tkg 1c3i 1hgt 1nc3 1ro7 1xo2 2cm8 2j7d 2q95 2weh 2ze1 3db6 3ghe 3kr5 3og7 3rx8 3uwl 4avw 4drp 4hg7 4kil 4nie 4tki 1c3r 1hi3 1nc6 1ros 1xoe 2cma 2j7e 2q96 2wei 2zfi 3db8 3gi4 3kr8 3ogm 3rxa 3uwo 4aw5 4dru 4hgc 4kin 4nj3 4tkn 1c3x 1hi4 1nd5 1rp7 1xog 2cmb 2j7f 2q9m 2wej 2zfp 3dba 3gi5 3krd 3ogp 3rxb 3ux0 4aw8 4ds1 4hge 4kio 4nk9 4tln 1c4u 1hi5 1nde 1rpa 1xom 2cmc 2j7g 2q9n 2weo 2zfs 3dbd 3gi6 3krj 3ogq 3rxd 3uxd 4awf 4dst 4hgl 4kip 4nka 4tlr 1c4v 1hih 1ndj 1rpf 1xon 2cmf 2j7h 2q9y 2weq 2zft 3dbs 3gjq 3krl 3ogx 3rxe 3uxg 4awg 4dsu 4hgs 4kiq 4nks 4tmk 1c4y 1hii 1ndv 1rpj 1xoq 2cmo 2j7w 2qa8 2wer 2zg1 3dbu 3gjs 3krr 3ohf 3rxf 3uxk 4awi 4dsy 4hgt 4kiu 4nkt 4tmn 1c50 1him 1ndw 1rq2 1xor 2cn0 2j7x 2qaf 2wev 2zg3 3dc2 3gjt 3krw 3ohh 3rxg 3uxl 4awj 4dt2 4hhy 4kju 4nku 4tmp 1c5c 1hiv 1ndy 1rql 1xos 2cn8 2j83 2qbp 2wey 2zga 3dc3 3gjw 3krx 3ohi 3rxh 3uxm 4awk 4dt6 4hhz 4kjv 4nl1 4tn2 1c5f 1hiy 1ndz 1rr6 1xot 2cne 2j87 2qbq 2wez 2zgm 3dcc 3gk1 3kry 3oik 3rxi 3uyr 4awm 4dtk 4hiq 4klb 4nld 4tnw 1c5n 1hk1 1nf8 1rri 1xow 2cnf 2j94 2qbr 2wf0 2zgx 3dcq 3gk2 3ksq 3oil 3rxj 3uyt 4awo 4dtt 4his 4kln 4nmo 4to8 1c5o 1hk2 1nfs 1rrw 1xoz 2cng 2j95 2qbs 2wf1 2zhd 3dcr 3gk4 3ktr 3oim 3rxk 3uz5 4awp 4du8 4hj2 4klv 4nmp 4tpk 1c5p 1hk3 1nfu 1rry 1xp0 2cnh 2j9a 2qbu 2wf2 2zif 3dcs 3gkz 3kv2 3oj8 3rxl 3uza 4awq 4duh 4hki 4km0 4nmq 4tpm 1c5q 1hk4 1nfw 1rs2 1xp1 2cni 2j9h 2qbw 2wf3 2zir 3dct 3gl6 3kvw 3ok9 3rxm 3uzc 4ax9 4dum 4hkk 4km2 4nmr 4tpp 1c5s 1hk5 1nfx 1rs4 1xp6 2co0 2j9l 2qbx 2wf5 2zis 3dcv 3gm0 3kvx 3oka 3rxo 3uzd 4axa 4dus 4hkn 4kmd 4nms 4tpt 1c5t 1hkj 1nfy

1rsd 1xp9 2csm 2j9n 2qc6 2wfg 2zit 3dcw 3gn7 3kw9 3okh 3rxp 3uzj 4axd 4dv8 4hkp 4kmu 4nmt 4tpw 1c5x 1hkk 1ngw 1rsi 1xpc 2csn 2ja3 2qcd 2wfj 2zjf 3dd0 3gnv 3kwa 3okp 3ry8 3uzp 4axm 4dv9 4hl5 4kmz 4nmv 4tq3 1c5y 1hkm 1nh0 1rst 1xpz 2ctc 2jaj 2qcf 2wgi 2zju 3dd8 3gnw 3kwb 3oku 3ryj 3v01 4ay5 4dve 4hla 4kn0 4nmx 4ts1 1c5z 1hlf 1nhg 1rt1 1xq0 2cv3 2jal 2qcg 2wgj 2zjv 3dda 3gol 3kwf 3okv 3ryv 3v04 4ay6 4dvf 4hlc 4kn1 4nnr 4tsx 1c6y 1hlk 1nhu 1rt2 1xqc 2cvd 2jb5 2qch 2wgs 2zjw 3ddb 3gp0 3kwj 3ole 3ryw 3v0p 4ayp 4dvi 4hld 4kn2 4no9 4tsz 1c70 1hmr 1nhv 1rt9 1xr8 2d06 2jb6 2qci 2who 2zkj 3ddf 3gpe 3kwn 3olf 3ryx 3v0y 4ayq 4dwb 4hlf 4kn4 4non 4tt2 1c7e 1hms 1nhw 1rtf 1xr9 2d0k 2jbj 2qcm 2whp 2zlf 3ddg 3gpj 3kwz 3olg 3ryy 3v1r 4ayr 4dwg 4hlg 4kn7 4np2 4tte 1c7f 1hmt 1nhx 1rth 1xsc 2d1n 2jbk 2qcn 2wi1 2zlg 3ddp 3gpo 3kx1 3oli 3ryz 3v2n 4ayt 4dwk 4hlh 4knb 4np3 4tu4 1c83 1hn2 1nhz 1rti 1xt3 2d1o 2jbl 2qd6 2wi2 2zm1 3ddq 3gqo 3kxz 3omc 3rz0 3v2o 4ayu 4dx9 4hlk 4kne 4np9 4tuh 1c84 1hn4 1ni1 1rtl 1xt8 2d1x 2jbo 2qd7 2wi3 2zm3 3ddu 3gqz 3kyf 3omg 3rz1 3v2p 4ayv 4dxg 4hlm 4kni 4npv 4tun 1c85 1hnn 1nj1 1ru1 1xuc 2d2v 2jbp 2qd8 2wi4 2zmd 3deh 3gr2 3kyg 3omm 3rz3 3v2q 4ayw 4dxj 4hlw 4knj 4nra 4tv3 1c86 1hos 1nj5 1ru2 1xud 2d3u 2jbu 2qd9 2wi5 2zmj 3dei 3grj 3kyq 3oob 3rz5 3v2w 4ayx 4dy6 4hmh 4knm 4nrb 4tw6 1c87 1hp0 1nja 1rv1 1xug 2d3z 2jbv 2qdt 2wi6 2zmm 3dej 3gs6 3kyr 3oof 3rz7 3v2x 4ayy 4dzw 4hmk 4knr 4nrc 4tw7 1c88 1hp5 1njb 1rw8 1xuo 2d41 2jc0 2qe2 2wi7 2zn7 3dek 3gs7 3l08 3ook 3rz8 3v30 4az0 4dzy 4hmq 4knx 4nrk 4tw8 1c8l 1hpo 1njc 1rwq 1xur 2dbl 2jdh 2qe4 2wib 2zns 3dg8 3gsg 3l0e 3oot 3rz9 3v31 4az2 4e0w 4hn2 4ko8 4nrl 4tw9 1c8v 1hps 1njd 1rww 1xws 2ddf 2jdl 2qe5 2wic 2znt 3dga 3gsm 3l0k 3ooz 3rzb 3v3b 4az3 4e0x 4hnc 4kod 4nrm 4twc 1c9d 1hpv 1nje 1rwx 1xxh 2df6 2jdm 2qf6 2wig 2znu 3dgl 3gss 3l0n 3opm 3rzi 3v3l 4az5 4e1e 4hnf 4kom 4nro 4twd 1ca8 1hpx 1njf 1rxp 1xxi 2doo 2jdn 2qfo 2wih 2zo3 3dgn 3gst 3l0v 3opp 3s00 3v3m 4az6 4e1k 4hni 4kon 4nrp 4tx6 1caq 1hq5 1njj 1ryh 1xz8 2dq7 2jdo 2qft 2wij 2zoq 3dgo 3gt9 3l13 3oq5 3s0b 3v3q 4aza 4e1m 4hnn 4kot 4nrq 4txc 1cbr 1hqf 1njs 1rzx 1xzx 2drc 2jdp 2qfu 2wik 2zp0 3dgq 3gta 3l16 3oqf 3s0d 3v3v 4azb 4e1n 4hnp 4kov 4nrt 4txe 1cbx 1hqg 1njt 1s19 1y0l 2dri 2jds 2qg0 2wj1 2zpk 3dhk 3gtc 3l17 3oqk 3s0e 3v43 4azc 4e1z 4hod 4kow 4nru 4ty6 1ce5 1hqh 1nju 1s26 1y0x 2ds1 2jdt 2qg2 2wj2 2zq0 3dhv 3gur 3l1n 3orn 3s0j 3v49 4aze 4e20 4hp0 4kox 4ntj 4ty7 1cea 1hrn 1nkm 1s38 1y19 2dua 2jdv 2qh6 2wjg 2zq1 3di6 3gus 3l1s 3os3 3s0n 3v4j 4azg 4e26 4hpi 4kp0 4nuc 4tyl 1ceb 1hs6 1nl4 1s39 1y1m 2duv 2jdy 2qhc 2wji 2zq2 3diw 3guz 3l2y 3osi 3s0o 3v4t 4azi 4e28 4hpy 4kp4 4nud 4tyo 1cet 1hsg 1nl6 1s3k 1y1z 2dw7 2jew 2qhd 2wk2 2zsc 3djf 3gv6 3l38 3osw 3s1g 3v4v 4azp 4e34 4hra 4kp5 4nue 4tyt 1cgl 1hsl 1nl9 1s4d 1y20 2dwx 2jf4 2qhm 2wk6 2zu3 3djk 3gv9 3l3l 3ot3 3s1h 3v4x 4azt 4e35 4hs6 4kp6 4nuf 4tz2 1ch8 1htf 1nli 1s50 1y2a 2dxs 2jfh 2qhn 2wks 2zu4 3djo 3gvb 3l3m 3ot8 3s1y 3v51 4azy 4e3b 4hs8 4kp8 4nus 4tz8 1ci7 1htg 1nlj 1s5q 1y2b 2e1w 2jfz 2qhr 2wkt 2zu5 3djp 3gvu 3l3n 3otf 3s22 3v5g 4b00 4e3d 4hso 4kpx 4nvp 4tzm 1cil 1hti 1nlo 1s5z 1y2c 2e27 2jg0 2qhy 2wky 2zv2 3djq 3gw5 3l3q 3otq 3s2a 3v5j 4b05 4e3f 4ht0 4kpz 4nvq 4tzn 1cim 1hty 1nlt 1s63 1y2d 2e2b 2jg8 2qhz 2wkz 2zv9 3djv 3gws 3l3x 3otx 3s2o 3v5l 4b0c 4e3g 4ht2 4kql 4nw2 4tzq 1cin 1hv5 1nm6 1s64 1y2f 2e2p 2jgs 2qi0 2wl0 2zva 3djx 3gwt 3l3z 3ouh 3s2p 3v5p 4b0g 4e3h 4ht6 4kqo 4nw5 4u03 1ciz 1hvh 1nmk 1s89 1y2g 2e2r 2jh0 2qi1 2wl4 2zvj 3dk1 3gwu 3l4t 3oui 3s2v 3v5q 4b0j 4e3i 4htp 4kqp 4nw6 4u0a 1cj1 1hvi 1nms 1s9t 1y2h 2e5y 2jh5 2qi3 2wl5 2zwz 3dkf 3gwv 3l4u 3ouj 3s3i 3v5t 4b11 4e3j 4htx 4kqq 4nw7 4u0b 1cka 1hvj 1nnb 1s9v 1y2j 2e7f 2jh6 2qi4 2wly 2zx5 3dkg 3gww 3l4v 3ov1 3s3m 3v66 4b12 4e3l 4hu1 4kqr 4nwc 4u0c 1ckb 1hvk 1nnk 1sb1 1y2k 2e7l 2jiu 2qi5 2wlz 2zx7 3dkj 3gwx 3l4w 3ove 3s3n 3v6r 4b13 4e3m 4huo 4krs 4nwd 4u0d 1clu 1hvl 1nnu 1sbg 1y3a 2e91 2jiw 2qi6 2wm0 2zx8 3dla 3gx0 3l4y 3ovn 3s3o 3v6s 4b14 4e3n 4hup 4ks1 4nwk 4u0e 1cnw 1hvr 1nny 1sbr 1y3g 2e92 2jj3 2qi7 2wmu 2zx9 3dln 3gxl 3l4z 3ovx 3s3q 3v78 4b1c 4e3o 4hv3 4ks2 4nwm 4u0f 1cnx 1hvs 1no6 1sc8 1y3n 2e93 2jjb 2qic 2wmv 2zxa 3dm6 3gxt 3l54 3ovz 3s3r 3v7c 4b1d 4e49 4hv7 4ks3 4nxs 4u0g 1cny 1hvy 1no9 1sdt 1y3p 2e94 2jjk 2qju 2wmw 2zxb 3dnd 3gxy 3l58 3ow3 3s3v 3v7d 4b1j 4e4a 4hva 4ks4 4nxu 4u0i 1cp6 1hwr 1noi 1sdu 1y3v 2e95 2jjr 2qk5 2wn9 2zxd 3dne 3gxz 3l59 3ow4 3s43 3v7s 4b2d 4e4n 4hvb 4ks5 4ny3 4u0m 1cpi 1hww 1noj 1sdv 1y3w 2e98 2jk7 2qk8 2wnc 2zxg 3dng 3gy2 3l5b 3ow6 3s45 3v7t 4b2i 4e5d 4hvd 4ksp 4nyf 4u0n 1cps 1hxb 1nok 1sfi 1y3x 2e99 2jk9 2qki 2wnj 2zy1 3dnj 3gy3 3l5c 3owb 3s4q 3v7x 4b2l 4e5f 4hvg 4ksq 4nyi 4u0s 1cqp 1hxk 1nox 1sgu 1y3y

2e9a 2jke 2qky 2wnl 2zyb 3dnt 3gy4 3l5d 3owd 3s53 3v8s 4b32 4e5g 4hvh 4ksy 4nyj 4u0u 1cr6 1hxw 1np0 1sh9 1y4z 2e9d 2jkh 2ql5 2wo8 2zym 3dog 3gy7 3l5e 3owj 3s54 3v8t 4b33 4e5h 4hvi 4ktc 4nym 4u43 1cs4 1hy7 1npa 1shc 1y57 2e9n 2jkk 2ql7 2wo9 2zyn 3dow 3gyn 3l5f 3owl 3s56 3v8w 4b34 4e5i 4hvs 4ktu 4nyt 4u44 1csh 1hyo 1npv 1shd 1y6a 2e9o 2jkm 2qlb 2woa 2zz1 3doy 3gz9 3l5r 3own 3s5y 3v9b 4b35 4e5j 4hw2 4kup 4nzb 4u45 1csi 1hyv 1npw 1siv 1y6b 2e9u 2jko 2qlf 2won 2zz2 3doz 3gzn 3l6f 3oxc 3s68 3va4 4b3b 4e5w 4hw7 4kv9 4nze 4u4s 1csr 1hyz 1npz 1sj0 1y6q 2e9v 2jkp 2qlj 2woq 2zz6 3dp0 3h03 3l6h 3oxi 3s6t 3vb4 4b3c 4e6c 4hwb 4kva 4nzm 4u4x 1css 1i00 1nq0 1sje 1y6r 2ea2 2jkq 2qlm 2wor 2zzu 3dp1 3h06 3l6x 3oxz 3s71 3vb5 4b3d 4e6d 4hwo 4kvm 4nzn 4u5j 1ct8 1i1e 1nq7 1sjh 1y8o 2ea4 2jkr 2qln 2wos 3a1c 3dp2 3h0b 3l79 3oy0 3s72 3vb6 4b3u 4e6q 4hwp 4kw6 4nzo 4u6r 1ctr 1i2s 1nqc 1skj 1y8p 2eas 2jkt 2qlq 2wot 3a1d 3dp3 3h0e 3l7a 3oy1 3s73 3vb7 4b4g 4e70 4hwr 4kwf 4o04 4u6x 1ctt 1i32 1nt1 1sl3 1y91 2eep 2jld 2qm7 2wou 3a1e 3dp4 3h0j 3l7b 3oy3 3s74 3vbd 4b4m 4e7r 4hws 4kwg 4o05 4u6y 1ctu 1i33 1ntk 1sld 1y98 2eg7 2jle 2qm9 2wp1 3a1s 3dp9 3h0q 3l7c 3oy8 3s75 3vbg 4b4n 4e81 4hwt 4kwo 4o07 4u79 1cw2 1i37 1ntv 1sle 1ya4 2eg8 2jlo 2qmd 2wpa 3a1t 3dpc 3h0s 3l7d 3oyl 3s76 3vbq 4b4q 4e8w 4hww 4kwp 4o0a 4u7t 1cwb 1i3z 1nu1 1slg 1ybg 2eh8 2jmj 2qmf 2wpb 3a29 3dpd 3h0v 3l7g 3oyn 3s77 3vbt 4b5b 4e8y 4hxj 4kww 4o0b 4u8w 1cwc 1i41 1nu3 1sln 1ybo 2ei6 2jnp 2qmg 2wq4 3a2c 3dpe 3h0w 3l81 3oyp 3s78 3vbv 4b5d 4e8z 4hxl 4kx8 4o0j 4u90 1cx9 1i43 1nu8 1sm2 1yc1 2emt 2jnw 2qmj 2wq5 3a2o 3dpf 3h0y 3l8s 3oys 3s7a 3vbw 4b5s 4e90 4hxm 4kxb 4o0r 4u91 1cyn 1i48 1nvq 1sm3 1yc4 2epn 2joa 2qn1 2wqb 3a3y 3dpk 3h0z 3l8v 3oyw 3s7b 3vbx 4b5t 4e93 4hxq 4kxl 4o0t 4u93 1czc 1i5d 1nvr 1sme 1yc5 2er0 2jq9 2qn2 2wr8 3a4o 3dpo 3h1x 3l8x 3oz1 3s7f 3vby 4b5w 4e96 4hxr 4kxm 4o0v 4ua8 1cze 1i5h 1nvs 1snk 1yci 2er6 2jqi 2qn3 2wsx 3a4p 3drf 3h1z 3l9h 3ozg 3s7l 3vc4 4b60 4e9c 4hxs 4kxn 4o0x 4uac 1czk 1i5r 1nw4 1so2 1ycm 2er9 2jqk 2qnn 2wtc 3a5y 3drg 3h21 3l9l 3ozj 3s7m 3vd4 4b6c 4e9d 4hxw 4kyh 4o0y 4ual 1czl 1i6v 1nw5 1soj 1yda 2erz 2jql 2qnp 2wtd 3a6t 3dri 3h22 3l9m 3ozp 3s8l 3vd7 4b6e 4e9u 4hxz 4kyk 4o0z 4uat 1czo 1i72 1nw7 1sps 1ydb 2esm 2jsd 2qnq 2wti 3a73 3drp 3h23 3l9n 3ozr 3s8n 3vd9 4b6f 4ea1 4hy0 4kz0 4o10 4uau 1czq 1i7c 1nwl 1sqa 1ydd 2etk 2jst 2qnx 2wtj 3a9i 3drr 3h26 3lau 3ozs 3s8o 3vdb 4b6o 4ea2 4hy1 4kz3 4o12 4um9 1czr 1i7g 1nxy 1sqb 1ydk 2etm 2jt5 2qnz 2wtv 3aaq 3drs 3h2a 3lbj 3ozt 3s8x 3vdc 4b6p 4ea3 4hy4 4kz4 4o13 4uma 1d04 1i7i 1ny0 1sqc 1ydr 2etr 2jup 2qo1 2wtw 3aas 3ds0 3h2c 3lbk 3p0g 3s9e 3veh 4b6q 4ear 4hy5 4kz5 4o15 4umb 1d09 1i7m 1ny2 1sqi 1yds 2euf 2jxr 2qo8 2wtx 3aau 3ds1 3h2f 3lbl 3p17 3s9i 3veu 4b6r 4eb8 4hy9 4kz6 4o1b 4umc 1d1p 1i7z 1nym 1sqn 1yei 2euk 2k00 2qoa 2wu6 3aav 3ds4 3h2m 3lbz 3p1d 3s9t 3vf3 4b6s 4eb9 4hyb 4kz7 4o1d 4umn 1d2e 1i80 1nyx 1sqo 1yej 2eum 2k0g 2qoe 2wu7 3abt 3ds6 3h2n 3lc3 3p23 3s9y 3vf5 4b6u 4ebs 4hyf 4kz8 4o1l 4umq 1d2s 1i8h 1nyy 1sqp 1yet 2evc 2k0x 2qoh 2wuf 3abu 3dst 3h2o 3lc5 3p2e 3s9z 3vf7 4b70 4ebt 4hyh 4kza 4o24 4umr 1d3d 1i8i 1nz7 1sqq 1yfz 2evl 2k1q 2qp6 2wuu 3acl 3dsu 3h30 3lcd 3p2h 3sad 3vf8 4b71 4ebv 4hyi 4kzb 4o28 4umt 1d3p 1i8j 1nzl 1sqt 1yhm 2evm 2k2g 2qp8 2wva 3acw 3dsz 3h3c 3lce 3p2k 3sap 3vf9 4b72 4ec0 4hym 4kzc 4o2a 4umu 1d3q 1i8z 1nzq 1sr7 1yhs 2evo 2k2r 2qpj 2wvt 3acx 3dt1 3h52 3lco 3p3g 3sax 3vfa 4b73 4ec4 4hys 4kzl 4o2c 4uof 1d3v 1i90 1nzv 1sre 1yi3 2ew5 2k31 2qpq 2wvz 3ad7 3dtc 3h59 3lcu 3p3j 3saz 3vfb 4b74 4ede 4hyu 4kzq 4o2e 4uoh 1d4h 1i91 1o0d 1srg 1yid 2ew6 2k3w 2qpu 2ww0 3ad8 3du8 3h5b 3lcv 3p3r 3sb0 3vfj 4b76 4edu 4hz5 4kzu 4o2f 4up5 1d4i 1i9l 1o0f 1sri 1yk7 2ewa 2k46 2qq7 2ww2 3ads 3dux 3h5s 3ldp 3p3s 3sbh 3vfq 4b77 4edy 4hze 4l02 4o36 4url 1d4j 1i9m 1o0h 1ssq 1ykp 2ewb 2k4i 2qqs 2wwj 3adt 3duy 3h5u 3ldq 3p3t 3sc1 3vg1 4b78 4edz 4hzm 4l09 4o37 4urm 1d4k 1i9n 1o0m 1stc 1ykr 2ewp 2k62 2qrg 2wxd 3adu 3dv1 3h6z 3ldw 3p3u 3sd5 3vgc 4b7j 4ee0 4hzt 4l0b 4o3a 4urn 1d4l 1i9o 1o0n 1std 1ylv 2ews 2k7l 2qrh 2wxf 3adv 3dv5 3h78 3le6 3p44 3sdg 3vh9 4b7n 4eeh 4hzw 4l0i 4o3b 4uro 1d4p 1i9p 1o0o 1stp 1yly 2ewy 2kaw 2qrk 2wxh 3afk 3dvp 3h85 3le8 3p4f 3sdi 3vha 4b7p 4eej 4hzx 4l0l 4o3c 4us3 1d4t 1i9q 1o1s 1str 1ym1 2exc 2kbr 2qrl 2wxi 3ag9 3dwb 3h89 3le9 3p4r 3sdk 3vhc 4b7r 4eev 4hzz 4l0s 4o3f 4us4 1d4w 1iau 1o2g 1sts 1ym2 2exg 2kbs 2qrm 2wxj 3agl 3dx0 3h8b 3lea 3p4v 3sfc 3vhd 4b7z 4ef4 4i06 4l0t 4o3t 4usi 1d4y 1ibc 1o2h 1sv3 1ym4 2exm 2kce 2qrp 2wxk 3agm 3dx1 3h8c 3lf0 3p4w 3sff 3vhe 4b80 4ef6 4i0d 4l0v 4o3u 4usj 1d5j 1ibg 1o2j 1sve 1yms 2ez5 2kdh 2qrq 2wxl 3ah8 3dx2 3h91 3lfn 3p50 3sfg 3vhk 4b81 4efg 4i0f 4l10 4o42 4usw 1d6n 1icj 1o2k 1svg 1ymx 2ez7 2ke1 2qry

2wxn 3ahn 3dx3 3h98 3lfs 3p55 3sfh 3vhu 4b82 4efk 4i0r 4l19 4o43 4utn 1d6s 1ida 1o2n 1svh 1ynd 2f01 2kff 2qt5 2wxo 3aho 3dx4 3h9f 3lgl 3p58 3sfi 3vhv 4b83 4efs 4i0s 4l1a 4o44 4utr 1d6v 1idb 1o2o 1sw1 1yon 2f0z 2kfg 2qt9 2wxp 3ai8 3dxg 3h9k 3lgp 3p5k 3sgt 3vi2 4b84 4eft 4i0t 4l1u 4o45 4utv 1d6w 1idg 1o2p 1sw2 1you 2f10 2kfh 2qta 2wxq 3aid 3dxh 3h9o 3lgs 3p5l 3sgv 3vi5 4b85 4efu 4i0z 4l23 4o4g 4utx 1d7i 1ie9 1o2q 1swg 1yp9 2f14 2kfx 2qtb 2wxv 3aig 3dxj 3ha6 3lhg 3p5o 3sgx 3vi7 4b8o 4eg4 4i10 4l2f 4o4k 4uu7 1d7j 1iem 1o2r 1swi 1ype 2f18 2kgi 2qtg 2wyf 3aje 3dxm 3hab 3lhj 3p76 3sh0 3vid 4b8p 4eg6 4i11 4l2g 4o4r 4uu8 1d7x 1iep 1o2s 1swk 1ypg 2f1a 2khh 2qtn 2wyg 3al3 3dy6 3hau 3lhs 3p78 3sh1 3vjc 4b8y 4eg7 4i12 4l2l 4o4y 4uua 1d8e 1iew 1o2t 1swn 1ypj 2f1b 2kmx 2qtr 2wyi 3alt 3dya 3hav 3li2 3p79 3sha 3vje 4b95 4ega 4i1c 4l2x 4o55 4uub 1d8f 1if7 1o2u 1swp 1yq7 2f1g 2knh 2qtt 2wyj 3ama 3dyo 3hb4 3lik 3p7a 3shb 3vjk 4b9h 4egh 4i2w 4l31 4o5b 4uv8 1d8m 1if8 1o2v 1swr 1yqj 2f2c 2ko7 2qtu 2wyn 3amb 3dz2 3hb8 3lil 3p7b 3shc 3vjl 4b9w 4egi 4i2z 4l32 4o5g 4uv9 1d9i 1igb 1o2w 1syh 1yqy 2f2h 2koh 2qu2 2wzf 3amv 3dz4 3hbo 3lir 3p7c 3shj 3vjm 4b9z 4egk 4i31 4l33 4o61 4uva 1dar 1igj 1o2x 1syi 1yrs 2f34 2kp8 2qu3 2wzm 3anq 3dz5 3hc8 3liw 3p7i 3shv 3vjs 4ba3 4eh2 4i32 4l34 4o6e 4uvb 1db1 1ih0 1o2y 1syo 1ysg 2f35 2kpl 2qu5 2wzs 3anr 3dz6 3hcm 3lj3 3p8e 3shy 3vjt 4bae 4eh3 4i33 4l3o 4o6w 4uvc 1db4 1ihy 1o2z 1sz0 1ysi 2f3e 2krd 2qu6 2wzx 3ant 3dzt 3hd3 3lj7 3p8h 3shz 3vnt 4bah 4eh4 4i3z 4l3p 4o70 4uwf 1db5 1ii5 1o30 1szd 1yt9 2f3f 2ks9 2qv7 2wzy 3ao1 3e01 3hdk 3ljg 3p8n 3si3 3vo3 4bak 4eh5 4i47 4l4l 4o71 4uwg 1dbb 1iig 1o32 1szm 1yuc 2f3k 2ksa 2qve 2wzz 3ao2 3e0p 3hdm 3ljj 3p8o 3si4 3voz 4bam 4eh6 4i4e 4l4m 4o72 4uwh 1dbj 1iih 1o33 1t08 1yvh 2f3r 2ksb 2qw1 2x00 3ao4 3e0q 3hdn 3ljo 3p8p 3sie 3vp1 4ban 4eh7 4i4f 4l4v 4o75 4uwk 1dbk 1iiq 1o34 1t13 1yvm 2f4b 2ksp 2qwb 2x09 3ao5 3e12 3hdz 3ljt 3p8z 3sio 3vp2 4bao 4eh8 4i54 4l4z 4o76 4uwl 1ddm 1ijr 1o35 1t1r 1yvz 2f4j 2kup 2qwc 2x0y 3aox 3e16 3hec 3ljz 3p9h 3sjf 3vp3 4baq 4eh9 4i5c 4l50 4o77 4ux6 1det 1ik4 1o36 1t1s 1yw2 2f5t 2kvm 2qwd 2x24 3ap7 3e1r 3heg 3lk0 3p9j 3sji 3vp4 4bb2 4ehe 4i5h 4l51 4o78 4uxq 1df8 1ikt 1o37 1t29 1yw7 2f6j 2kwn 2qwe 2x2c 3apc 3e2m 3hek 3lk1 3p9l 3sjo 3vqh 4bb4 4ehg 4i5m 4l52 4o7a 4uy1 1dfo 1ikv 1o38 1t2v 1yw8 2f6t 2kzu 2qwf 2x2i 3aqa 3e37 3hf6 3lk8 3p9m 3sjt 3vqs 4bb9 4ehm 4i5p 4l53 4o7b 4uyd 1dg9 1ikw 1o39 1t31 1ywh 2f6v 2l0i 2qwg 2x2k 3aqt 3e3b 3hf8 3lka 3p9t 3sk2 3vqu 4bbe 4ehr 4i60 4l58 4o7c 4uye 1dgm 1ikx 1o3b 1t32 1ywi 2f6y 2l11 2qx0 2x2l 3ara 3e3c 3hfb 3lkh 3pa3 3ska 3vrt 4bbf 4ehv 4i67 4l5j 4o7e 4uyf 1dhi 1iky 1o3c 1t37 1ywr 2f6z 2l12 2qyk 2x2m 3arb 3e3u 3hfj 3lkz 3pa4 3skc 3vru 4bbg 4ehz 4i6b 4l6s 4o7f 4uyg 1dhj 1il3 1o3d 1t3t 1yxd 2f70 2l1b 2qyl 2x2r 3ard 3e4a 3hfv 3ll8 3pa5 3ske 3vrv 4bbh 4ei4 4i6f 4l6t 4o91 4uyh 1di8 1il4 1o3f 1t46 1yy4 2f71 2l1r 2qyn 2x38 3arf 3e51 3hha 3lle 3pa8 3skf 3vrw 4bc5 4ej2 4i6h 4l70 4o97 4uyn 1di9 1il5 1o3g 1t48 1yy6 2f7i 2l3r 2qzk 2x39 3arg 3e5a 3hhk 3lm1 3pab 3skg 3vry 4bcb 4ej8 4i6q 4l7b 4o9s 4uzd 1dif 1il9 1o3h 1t49 1yye 2f7o 2l65 2qzl 2x3t 3arn 3e5u 3hhm 3lmk 3pax 3skh 3vs3 4bcc 4ejf 4i71 4l7c 4o9v 4uzh 1dis 1ilh 1o3i 1t4e 1yyr 2f80 2l6e 2qzr 2x4o 3arp 3e62 3hhu 3lmp 3pb3 3skk 3vsw 4bcd 4ejl 4i72 4l7d 4o9w 4v01 1dkd 1ilq 1o3j 1t4j 1yys 2f81 2l6j 2qzx 2x4r 3arr 3e63 3hig 3lnj 3pb7 3sl0 3vsx 4bcf 4ejn 4i73 4l7f 4oag 4v04 1dl7 1imx 1o3k 1t4s 1yyy 2f89 2l75 2r02 2x4s 3art 3e64 3hii 3lnk 3pb8 3sl1 3vtb 4bcg 4ek9 4i74 4l7g 4oak 4v05 1dm2 1inc 1o3l 1t4v 1z1h 2f8g 2l7u 2r03 2x4t 3aru 3e6k 3hik 3lnz 3pb9 3sl4 3vtc 4bch 4eke 4i7b 4l7h 4oar 4v1c 1dmb 1ind 1o3p 1t5a 1z1r 2f8i 2l84 2r05 2x4u 3arv 3e6v 3hio 3lok 3pbb 3sl5 3vtd 4bci 4ekg 4i7c 4l7j 4oas 4v24 1dmp 1inf 1o41 1t5f 1z2b 2f94 2l8j 2r0h 2x4z 3arw 3e6y 3hit 3loo 3pcb 3sl8 3vtr 4bcj 4eki 4i7d 4l7l 4oba 4v25 1dmt 1ing 1o42 1t69 1z34 2f9b 2l8r 2r0u 2x5o 3arx 3e73 3hiv 3lox 3pcc 3slz 3vuc 4bck 4eky 4i7f 4l7n 4obo 4v27 1doj 1inh 1o43 1t79 1z3c 2f9k 2l98 2r0y 2x6d 3ary 3e7a 3hiw 3lp0 3pce 3sm0 3vv6 4bcm 4el0 4i7j 4l7o 4obp 4w4z 1dpu 1inq 1o44 1t7d 1z3j 2f9u 2las 2r0z 2x6e 3arz 3e7b 3hj0 3lp1 3pcf 3sm1 3vv7 4bcn 4el5 4i7k 4l7r 4obq 4w50 1dqn 1iq1 1o45 1t7f 1z3t 2f9v 2lbm 2r1w 2x6f 3as0 3e7o 3hjo 3lp2 3pcg 3sm2 3vv8 4bco 4el9 4i7l 4l7u 4obv 4w5j 1dqx 1irs 1o46 1t7j 1z3v 2fah 2lbv 2r1x 2x6i 3as1 3e81 3hk1 3lp4 3pch 3smq 3vva 4bcp 4elb 4i7m 4l8m 4obz 4w7p 1drj 1is0 1o47 1t7r 1z4n 2fai 2lcs 2r23 2x6j 3as2 3e85 3hkn 3lp7 3pcj 3sn7 3vvy 4bcq 4elc 4i7p 4l9i 4oc0 4w7t 1drk 1it6 1o48 1ta2 1z4o 2fb8 2lct 2r2b 2x6k 3as3 3e8n 3hkq 3lpb 3pck 3sn8 3vvz 4bcs 4ele 4i80 4la7 4oc1 4w97 1drv 1iup 1o49 1ta6 1z4u 2fci 2lgf 2r2l 2x6w 3ask 3e8r 3hkt 3lpf 3pcn 3sna 3vw0 4bcw 4elf 4i8n 4lar 4oc2 4w9c 1dth 1ivp 1o4a 1tbz 1z5m 2fda 2lh8 2r2m 2x6x 3asl 3e8u 3hku 3lpg 3pcu 3snb 3vw1 4bd3 4elh 4i8w 4lbl

4oc3 4w9d 1dtq 1iwq 1o4d 1tc1 1z6d 2fdd 2lha 2r2w 2x6y 3asx 3e90 3hkw 3lpi 3pd2 3snc 3vw2 4bda 4em7 4i8x 4lbo 4oc4 4w9e 1dtt 1iy7 1o4e 1tcw 1z6e 2fde 2liq 2r38 2x7d 3at1 3e92 3hky 3lpj 3pd3 3snd 3vw6 4bdb 4emf 4i8z 4lbp 4oc5 4w9f 1dub 1iyl 1o4f 1tcx 1z6f 2fdp 2lk1 2r3c 2x7o 3at3 3e9b 3hl5 3lpk 3pd4 3sni 3vw7 4bdc 4emr 4i9c 4lbu 4oc6 4w9g 1dud 1izh 1o4g 1td7 1z6j 2feq 2lkk 2r3f 2x7s 3at4 3e9h 3hl7 3lpl 3pd8 3snl 3vw9 4bdd 4emt 4i9h 4lc7 4occ 4w9h 1duv 1izi 1o4i 1tet 1z6p 2fes 2lko 2r3g 2x7t 3ati 3e9i 3hl8 3lpp 3pd9 3so6 3vws 4bde 4emv 4i9i 4lch 4ock 4w9i 1dva 1j01 1o4j 1tfq 1z6q 2ff1 2ll6 2r3h 2x7u 3atk 3eax 3hll 3lpr 3pdc 3so9 3vx3 4bdf 4en4 4i9o 4led 4ocp 4w9j 1dwc 1j07 1o4k 1tfz 1z6s 2ff2 2llo 2r3i 2x7x 3atl 3eb1 3hlo 3lpt 3pdh 3soq 3vyd 4bdg 4enx 4i9r 4leg 4ocq 4w9k 1dwd 1j14 1o4l 1tg5 1z71 2fgh 2llq 2r3j 2x81 3atm 3ebb 3hmm 3lpu 3pdq 3sou 3vye 4bdh 4eny 4i9s 4leq 4ocv 4w9l 1dx6 1j15 1o4m 1thl 1z95 2fgi 2lnw 2r3k 2x85 3atp 3ebh 3hmo 3lq2 3pe1 3sov 3vyf 4bdi 4eo4 4i9u 4lge 4ocx 4w9n 1dxp 1j16 1o4n 1thr 1z9g 2fgu 2lo6 2r3l 2x8d 3atu 3ebi 3hmp 3lq4 3pe2 3sow 3vzd 4bdj 4eo6 4i9z 4lgg 4ocz 4w9o 1dy4 1j17 1o4o 1ths 1z9h 2fgv 2loz 2r3m 2x8e 3atv 3ebl 3hmv 3lq5 3peq 3spf 3vzg 4bdk 4eo8 4ia0 4lgh 4od0 4w9p 1dzj 1j19 1o4p 1tjp 1z9y 2fhy 2lp8 2r3n 2x8i 3atw 3ebo 3hnb 3lq8 3pfp 3spk 3vzv 4bds 4eoh 4iaw 4lgu 4od7 4w9q 1dzk 1j1a 1o4q 1tka 1zaf 2fie 2lpr 2r3o 2x8z 3au6 3ebp 3hng 3lqi 3pgl 3sqq 3w07 4bdt 4eoi 4iax 4lh2 4od9 4w9s 1dzm 1j36 1o4r 1tkb 1zaj 2fix 2lsk 2r3p 2x91 3av9 3ecn 3hnz 3lqj 3pgu 3srb 3w0l 4bea 4eok 4ibb 4lh3 4ode 4waf 1dzp 1j37 1o5a 1tkc 1zc9 2fj0 2lsp 2r3t 2x95 3ava 3ed0 3ho2 3lrh 3phe 3src 3w1f 4bek 4eol 4ibc 4lh5 4odf 4wbo 1e00 1j4k 1o5b 1tkt 1zd2 2fjm 2lsr 2r3w 2x96 3avb 3ee2 3ho9 3ls4 3pi5 3srg 3w2o 4bf1 4eon 4ibd 4lh6 4oee 4wcu 1e02 1j4p 1o5c 1tkx 1zd3 2fjn 2lsv 2r3y 2x97 3avf 3eeb 3hp2 3luo 3pix 3srv 3w2p 4bf6 4eop 4ibe 4lh7 4oef 4wgi 1e03 1j4q 1o5e 1tkz 1zd4 2fjp 2lto 2r43 2x9e 3avg 3efj 3hp5 3lvp 3piy 3st5 3w2q 4bfd 4eor 4ibf 4lhm 4oeg 4wh7 1e06 1j4r 1o5f 1tl1 1zd5 2fkf 2ltv 2r4b 2x9f 3avh 3efk 3hp9 3lvw 3piz 3st6 3w2r 4bfp 4eos 4ibg 4lhv 4oeu 4wh9 1e1v 1j5i 1o5g 1tl3 1zdp 2fky 2ltw 2r4f 2xa4 3avi 3efr 3hpt 3lw0 3pj1 3std 3w2s 4bfr 4eox 4ibi 4li0 4ofl 4whh 1e1x 1j7z 1o5m 1tl7 1ze8 2fl2 2ltx 2r58 2xab 3avj 3efs 3hq5 3lxe 3pj2 3stj 3w2t 4bfz 4eoy 4ibj 4li5 4og3 4whl 1e1y 1j80 1o5p 1tl9 1zea 2fl5 2lty 2r59 2xae 3avk 3eft 3hqh 3lxg 3pj3 3str 3w32 4bg1 4ep2 4ibk 4li6 4og4 4whq 1e2k 1j81 1o5r 1tlo 1zeo 2fl6 2ltz 2r5a 2xag 3avl 3efw 3hqr 3lxk 3pj8 3su0 3w33 4bg6 4epy 4ibm 4li7 4og5 4whr 1e34 1jak 1o6h 1tlp 1zfk 2flb 2lwi 2r5b 2xah 3avm 3eg6 3hqw 3lxl 3pjc 3su1 3w37 4bgg 4eqc 4idn 4li8 4og6 4whs 1e37 1jao 1o6i 1tmb 1zfp 2fle 2ly0 2r5d 2xaj 3avn 3egk 3hqy 3lxo 3pjg 3su2 3w54 4bgh 4eqf 4ido 4lil 4og7 4wht 1e3g 1jaq 1o6q 1tmm 1zfq 2flh 2lya 2r5p 2xaq 3avz 3egt 3hqz 3lxs 3pjt 3su4 3w55 4bgk 4eqj 4idt 4lj3 4og8 4why 1e3v 1jbd 1o6r 1tmn 1zgb 2flr 2lyb 2r5q 2xas 3aw0 3ehn 3hr1 3ly2 3pju 3su5 3w5e 4bgm 4er1 4idv 4lj5 4ogi 4whz 1e4h 1jcx 1o79 1tng 1zge 2flu 2lyw 2r64 2xb7 3ax5 3eht 3hrb 3lzb 3pka 3su6 3w5n 4bgx 4er2 4idz 4lj8 4ogj 4wiv 1e55 1jd0 1o7o 1tnh 1zgi 2fm0 2lzg 2r6f 2xb8 3axk 3ehw 3hrf 3lzs 3pkb 3sud 3w5t 4bgy 4ere 4ie0 4ljh 4ogn 4wj5 1e5j 1jd5 1o86 1tni 1zgv 2fm2 2m0o 2r6n 2xb9 3axm 3ehx 3hs4 3lzu 3pkd 3sue 3w69 4bh3 4erf 4ie3 4lk6 4ogt 4wj7 1e66 1jdj 1o8b 1tnj 1zh7 2fm5 2m0u 2r6w 2xba 3axz 3ehy 3hs8 3lzv 3pke 3suf 3w9k 4bh4 4erk 4ie4 4lk7 4ogv 4wk2 1e6q 1jet 1o9d 1tnk 1zhk 2fmb 2m0v 2r6y 2xbj 3ay0 3eid 3hs9 3lzz 3pkn 3sug 3w9r 4bhf 4erq 4ie5 4lkd 4ohk 4wk7 1e6s 1jeu 1o9e 1tnl 1zhl 2fme 2m3m 2r75 2xbp 3ay9 3eig 3hu1 3m11 3plu 3sur 3wab 4bhn 4erw 4ie6 4lke 4ohm 4wke 1e72 1jev 1o9k 1tog 1zhy 2fo4 2m3o 2r7b 2xbv 3aya 3eio 3hu2 3m1j 3pm1 3sus 3wav 4bhz 4ery 4ie7 4lkf 4oho 4wki 1e8h 1jfh 1oai 1toj 1zkk 2fou 2m3z 2r7g 2xbw 3ayc 3ej1 3hu3 3m1k 3pma 3sut 3wax 4bi0 4erz 4ieh 4lkg 4ohp 4wks 1e9h 1jg0 1oar 1tok 1zkl 2fov 2mas 2r8q 2xbx 3ayd 3ej5 3hub 3m1s 3pn1 3suu 3way 4bi1 4es0 4ifi 4lkh 4ojq 4wlb 1eas 1jgl 1oau 1tom 1zkn 2foy 2mc1 2r9b 2xc0 3az8 3ejp 3huc 3m2u 3pn3 3suv 3wb4 4bi2 4esg 4igk 4lkj 4ojr 4wn1 1eat 1jh1 1oay 1tou 1zky 2fpz 2mg5 2r9m 2xc4 3az9 3ejq 3hv4 3m2w 3pn4 3suw 3wb5 4bi6 4esi 4igq 4lkk 4ok3 4wt2 1eb1 1jif 1oba 1tps 1zls 2fqo 2mip 2r9s 2xcg 3aza 3ejr 3hv5 3m35 3po1 3sv2 3wbl 4bi7 4est 4igr 4lkl 4ok5 4wvl 1eb2 1jii 1obx 1tpw 1zlu 2fqt 2mji 2r9w 2xch 3azb 3ejs 3hv6 3m36 3po6 3sv6 3wc5 4bib 4etz 4igt 4lkm 4ok6 4wwn 1ebg 1jij 1ocn 1tpz 1zlv 2fqw 2mkr 2r9x 2xck 3b0w 3eju 3hv7 3m37 3poa 3sv7 3wc7 4bic 4eu0 4ih3 4lko 4okg 4wwo 1ebw 1jik 1ocq 1tq4 1zlw 2fqx 2mnz 2ra0 2xcn 3b1m 3eka 3hv8 3m3c 3poz 3sv8 3wcb 4bid 4euc 4ih5 4lkq 4okp 4wwp 1eby 1jil 1od8 1tqf 1zm6 2fqy 2mov 2ra6 2xcs 3b1t 3ekn 3hvc 3m3e 3pp0 3sv9 3wcf 4bie 4euo 4ih6 4lkt 4oks 4wym 1ebz 1jiz 1odi

1tr7 1zm7 2fr3 2mow 2rc8 2xct 3b1u 3eko 3hvg 3m3o 3pp1 3svj 3wcg 4bio 4euv 4ih7 4ll3
4olc 4wz8 1ec0 1jj9 1odj 1trd 1zoe 2fr8 2mpa 2rc9 2xd6 3b24 3ekp 3hvh 3m3z 3pp7 3svv
3wch 4bis 4ew2 4iho 4llj 4old 4x7i 1ec1 1jje 1ody 1tsi 1zog 2frd 2mpm 2rcb 2xd9 3b25 3ekq
3hvi 3m40 3ppj 3sw2 3wci 4bj9 4ew3 4ii9 4llk 4olh 4yas 1ec2 1jjk 1oe0 1tsl 1zoh 2fs8 2mwo
2rcn 2xda 3b26 3ekr 3hvj 3m53 3ppk 3sw8 3wcl 4bjb 4ewh 4iic 4llp 4oma 5a3h 1ec3 1jjt
1oe7 1tsm 1zom 2fs9 2mwp 2rcu 2xde 3b27 3eks 3hvk 3m54 3ppm 3sw9 3wd1 4bjc 4ewn
4iid 4llx 4omc 5abp 1ec9 1jk7 1oe8 1tsv 1zp5 2fsa 2nm1 2rcw 2xdk 3b28 3ekt 3hw1 3m55
3ppo 3sww 3wd2 4bjx 4ewo 4iie 4llz 4ona 5apr 1ecq 1jld 1oeb 1tsy 1zp8 2fsv 2nmb 2rcx
2xdl 3b2q 3ekv 3hwn 3m56 3ppp 3sx4 3wd9 4bki 4ewr 4iif 4lm0 4onf 5er1 1ecv 1jlq 1ofz
1tt1 1zpa 2ftd 2nmx 2rd6 2xdm 3b2t 3ekw 3hww 3m57 3ppq 3sx9 3wdc 4bkj 4exg 4ij1 4lm1
4ono 5er2 1eed 1jlr 1ogd 1ttm 1zpb 2fts 2nmy 2reg 2xdw 3b2w 3ekx 3hwx 3m58 3pqz 3sxf
3wdd 4bks 4exh 4ijh 4lm2 4oo9 5est 1eef 1jlx 1ogg 1ttv 1zpc 2fu8 2nmz 2rf2 2xdx 3b3c 3eky
3hx3 3m59 3pr0 3sxu 3wde 4bkt 4exs 4ijl 4lm3 4oow 5fwg 1eei 1jm4 1ogu 1tu6 1zrz 2fum
2nn1 2rfh 2xe4 3b3s 3el0 3hxb 3m5a 3prf 3sym 3wdz 4bky 4exz 4ijp 4lm4 4op1 5hvp 1efi
1jmf 1ogx 1tuf 1zs0 2fuu 2nn7 2rfn 2xef 3b3w 3el1 3hxc 3m5e 3prs 3sz1 3we4 4bkz 4eyj 4ijq
4lm5 4op2 5lpr 1efy 1jmg 1ogz 1tv6 1zsb 2fv5 2nn8 2rfy 2xeg 3b3x 3el4 3hxd 3m67 3prz
3sz9 3wf5 4blb 4eym 4ikn 4lmn 4op3 5prc 1egh 1jmi 1oh4 1tve 1zsf 2fv9 2nnd 2rg5 2xei
3b4f 3el5 3hxe 3m6f 3ps1 3szb 3wf6 4bnt 4ez3 4ikr 4lmu 4oq3 5std 1eix 1jmq 1ohr 1tvo 1zsh
2fvc 2nng 2rg6 2xej 3b4p 3el7 3hxf 3m6p 3ps6 3szm 3wf7 4bnu 4ez5 4iks 4ln2 4oq5 5tln 1ej4
1jn2 1oi9 1tvr 1zsr 2fvd 2nnk 2rgp 2xel 3b50 3el8 3hxi 3m6q 3psb 3t01 3wf8 4bnv 4ezj 4ikt
4ln7 4oq6 5tmn 1ejn 1jn4 1oif 1tx7 1ztq 2fw3 2nno 2rgu 2xey 3b5j 3el9 3hy5 3m6r 3psd 3t03
3wf9 4bnx 4ezk 4iku 4lnb 4or0 5tmp 1ek1 1joc 1oim 1txr 1zub 2fw6 2nnp 2ri9 2xez 3b5r 3elc
3hy7 3m89 3psl 3t07 3wff 4bny 4ezl 4im0 4lnf 4oru 5upj 1ek2 1joj 1oiq 1tyn 1zuc 2fwp 2nnq
2ria 2xf0 3b65 3elj 3hy9 3m8p 3ptg 3t08 3wfg 4bnz 4ezo 4imq 4lng 4orx 5yas 1ekb 1jp5 1oir
1tyr 1zvx 2fwy 2nns 2rib 2xfi 3b66 3elm 3hyf 3m8q 3pty 3t09 3wgg 4bo0 4ezq 4imz 4lno
4ory 6abp 1ela 1jpl 1oit 1tys 1zxc 2fwz 2no3 2rin 2xfj 3b67 3emh 3hyg 3m8t 3puj 3t0d 3wgw
4bo1 4ezr 4in9 4lnp 4os1 6apr 1elb 1jq3 1oiu 1tze 1zxv 2fx6 2np8 2rio 2xg3 3b68 3eml 3hzk
3m8u 3puk 3t0l 3wha 4bo2 4ezt 4inb 4lnw 4os2 6cgt 1elc 1jq8 1oiy 1u0g 1zyj 2fx7 2np9 2rip
2xg5 3b78 3ens 3hzm 3m93 3pup 3t0m 3wi2 4bo3 4ezw 4inh 4loh 4os4 6cha 1eld 1jq9 1oj5
1u1b 1zyr 2fx8 2nq6 2rjp 2xg9 3b7i 3eoc 3hzv 3m94 3pvu 3t0t 3wi6 4bo4 4ezx 4inr 4loi 4os5
6cpa 1ele 1jqd 1ok7 1u1w 1zz1 2fx9 2nq7 2rjr 2xgm 3b7j 3eor 3hzy 3m96 3pvw 3t0w 3wig
4bo5 4ezy 4inu 4loj 4os6 6fiv 1elr 1jqe 1okl 1u2r 1zz2 2fxr 2nqg 2rjs 2xgo 3b7r 3eos 3i02
3m9f 3pwd 3t0x 3wix 4bo6 4ezz 4io2 4loo 4ot6 6gch 1els 1jqy 1oko 1u2y 1zz3 2fxs 2nqi 2rk8
2xgs 3b7u 3eou 3i06 3ma3 3pwh 3t19 3wiy 4bo7 4f08 4io3 4lop 4otg 6gpb 1em6 1jr1 1okv
1u32 1zzl 2fxu 2nsj 2rka 2xh5 3b82 3eov 3i0s 3mag 3pwk 3t1a 3wiz 4bo8 4f09 4io4 4loq 4oth
6lpr 1ent 1jrs 1okw 1u33 1zzz 2fxv 2nsl 2rkd 2xhm 3b8h 3eq7 3i1y 3mam 3pwm 3t1l 3wjw
4bo9 4f14 4io5 4lov 4oti 6prc 1enu 1jsv 1okx 1u3q 220l 2fys 2nsx 2rke 2xhr 3b8q 3eq8 3i25
3max 3pww 3t1m 3wk4 4bpi 4f1l 4io6 4loy 4otw 6rnt 1eoc 1jt1 1oky 1u3r 223l 2fyv 2nt7
2rkf 2xhs 3b8r 3eq9 3i28 3mb6 3px8 3t1n 3wk5 4bpj 4f1q 4io7 4lp0 4oty 6rsa 1eoj 1jtq 1ol1
1u3s 2a0c 2fzc 2nta 2rkg 2xht 3b8z 3eqb 3i3b 3mb7 3px9 3t2c 3wk6 4bps 4f1s 4io8 4lp6 4ou3
6std 1eol 1juf 1ol2 1u59 2a0t 2fzg 2ntf 2rkm 2xhx 3b92 3eql 3i3d 3mbl 3pxe 3t2p 3wk7 4bqg
4f20 4ipf 4lp9 4oue 6tim 1eou 1juj 1ols 1u65 2a14 2fzk 2nv7 2rkn 2xi7 3b95 3eqr 3i4a 3mct
3pxf 3t2q 3wk8 4bqh 4f2w 4ipi 4lpb 4ouj 6upj 1epo 1juq 1olu 1u6q 2a25 2g00 2nw4 2rku
2xib 3b9g 3eqs 3i4b 3mdz 3pxq 3t2t 3wk9 4bqs 4f39 4ipj 4lpf 4ov5 7abp 1epp 1jut 1olx 1u71
2a29 2g01 2nwl 2rl5 2xii 3b9s 3eqy 3i4y 3me9 3pxy 3t2v 3wka 4bqt 4f3c 4ipn 4lpg 4ovz 7cpa
1epq 1juy 1om1 1u8t 2a2g 2g0g 2nwn 2rly 2xix 3bar 3er3 3i51 3mea 3pxz 3t2w 3wkb 4bqw
4f3i 4iq6 4lph 4ow0 7gch 1erb 1jvp 1om2 1u9e 2a2x 2g0h 2nww 2rm0 2xiy 3bbb 3er5 3i5n
3met 3py0 3t3c 3wkc 4bqx 4f3k 4iqt 4lps 4owm 7gpb 1ero 1jvu 1om9 1u9l 2a31 2g19 2nxd
2rnw 2xiz 3bbf 3erd 3i5r 3meu 3py1 3t3d 3wkd 4bqy 4f49 4iqu 4lq3 4own 7hvp 1erq 1jwm
1ong 1u9q 2a3a 2g1q 2nxl 2rnx 2xj0 3bbt 3erk 3i5z 3mf5 3pyy 3t3e 3wke 4br3 4f4p 4irx 4lq9
4owo 7kme 1err 1jws 1onh 1u9v 2a3b 2g1r 2nxm 2rny 2xj1 3bc3 3ern 3i60 3mfv 3pz1 3t3g
3wmb 4brx 4f5y 4is6 4lqy 4owv 7lpr 1esz 1jwt 1onp 1u9w 2a3c 2g1y 2nyr 2rok 2xj2 3bc4
3ert 3i6c 3mfw 3pz2 3t3h 3wmc 4bs0 4f63 4ish 4lrh 4oya 7prc 1ets 1jwu 1ony 1u9x 2a3i 2g24
2o0u 2rol 2xj7 3bc5 3ery 3i6m 3mg0 3pz3 3t3i 3wp0 4bs4 4f65 4isi 4lrr 4oyb 7upj 1ett 1jyc

1onz 1ua4 2a3w 2g2r 2o1v 2rox 2xjg 3bcn 3esj 3i6o 3mg4 3pz4 3t3u 3wp1 4bs5 4f6v 4isu 4lsj 4oyg 830c 1etz 1jyi 1opi 1ucn 2a3x 2g5p 2o22 2rqu 2xjj 3bcs 3ess 3i6z 3mg6 3q0z 3t3v 3wqm 4bsq 4f6x 4ith 4lte 4oyi 8a3h 1eub 1jyq 1oq5 1udt 2a4l 2g5t 2o2u 2rr4 2xjx 3be2 3et7 3i73 3mg7 3q1x 3t3y 3wqv 4bt3 4f7v 4iti 4lts 4oyk 8abp 1evh 1jys 1oqp 1udu 2a4q 2g5u 2o3p 2rt5 2xk1 3be9 3eta 3i7b 3mhc 3q2a 3t4h 3wqw 4bt4 4f8h 4itj 4luo 4oym 8cpa 1ew8 1jzs 1ork 1uef 2a4r 2g63 2o3z 2sfp 2xk3 3bea 3eu5 3i7c 3mhi 3q2g 3t4n 3ws8 4bt5 4f8j 4itp 4luv 4oyo 8gpb 1ew9 1k03 1orw 1ugp 2a4w 2g6p 2o48 2sim 2xk4 3bel 3eu7 3i7e 3mhl 3q2h 3t4p 3ws9 4bt9 4f9g 4iu0 4luz 4oyp 8hvp 1ewj 1k06 1os0 1ugw 2a4z 2g6q 2o4h 2srt 2xk6 3bet 3evc 3i7g 3mhm 3q2j 3t4v 3wt5 4btb 4f9u 4iu1 4lv4 4oys 8lpr 1ex8 1k08 1os5 1ugx 2a58 2g70 2o4j 2std 2xk7 3bex 3evd 3i7i 3mho 3q2m 3t5i 3wt7 4bti 4f9v 4iu4 4lvt 4oyt 9abp 1exv 1k1i 1osg 1ugy 2a5b 2g71 2o4k 2tmn 2xk8 3bf1 3evf 3i81 3mhw 3q32 3t5u 3wv2 4btl 4f9w 4iue 4lw1 4oz1 9hvp 1exw 1k1j 1oss 1uh1 2a5c 2g72 2o4l 2toh 2xk9 3bft 3ew2 3i8t 3mi2 3q3b 3t60 3wyk 4btm 4f9y 4iur 4lwc 4oz2 9icd 1ez9 1k1l 1osv 1uho 2a5s 2g78 2o4n 2tpi 2xkc 3bfu 3ewc 3i90 3mi3 3q3k 3t64 3wyl 4btt 4fab 4iut 4lwt 4oz3 9lpr 1ezf 1k1m 1oth 1ui0 2a5u 2g79 2o4p 2tsr 2xkd 3bg8 3ewh 3i91 3miy 3q3t 3t6b 3wym 4btw 4fai 4iuu 4lwu 4ozj 1ezq 1k1n 1ouk 1uj0 2a8g 2g83 2o4r 2upj 2xke 3bgb 3ewj 3i97 3mj1 3q43 3t6j 3zbf 4btx 4fak 4iuv 4lwv 4ozl 1f0q 1k1o 1ouy 1uj5 2a8h 2g8n 2o4s 2usn 2xkf 3bgc 3ewu 3i9g 3mj2 3q44 3t6r 3zbx 4bty 4fbe 4iva 4lww 4ozn 1f0r 1k1p 1ov3 1uj6 2aa6 2g8r 2o4z 2uue 2xl2 3bgl 3ewz 3ia6 3mj5 3q4c 3t6y 3zc5 4bup 4fbx 4ivb 4lxa 4ozo 1f0s 1k1y 1ove 1ujj 2aa9 2g94 2o5d 2uuo 2xl3 3bgp 3ex2 3iae 3mjl 3q4j 3t70 3zc6 4buq 4fc0 4ivc 4lxb 4p00 1f0t 1k21 1ow4 1ujk 2aac 2g96 2o5k 2uup 2xlc 3bgq 3ex3 3iaf 3mke 3q4k 3t7g 3zcl 4bv2 4fcb 4ivd 4lxd 4p02 1f0u 1k22 1ow6 1uk0 2aay 2g97 2o63 2uw0 2xln 3bgz 3ex6 3iaw 3mkf 3q4l 3t82 3zcz 4bvb 4fcd 4ivk 4lxe 4p0a 1f1j 1k27 1ow7 1uk1 2ad5 2g9q 2o64 2uw3 2xm1 3bh3 3exe 3ibc 3mkn 3q5h 3t83 3zdg 4bw1 4fcf 4ivs 4lxk 4p0b 1f28 1k2i 1ow8 1ukh 2ada 2g9r 2o65 2uw4 2xm2 3bh8 3exh 3ibi 3ml2 3q5u 3t84 3zdh 4bw2 4fci 4ivt 4lxm 4p0n 1f2p 1k2v 1owd 1ukt 2adm 2g9u 2o7e 2uw5 2xm8 3bh9 3exo 3ibl 3ml4 3q6k 3t85 3zdv 4bw3 4fck 4iwd 4lxz 4p0v 1f3e 1k3n 1owe 1ule 2adu 2g9v 2o7n 2uw6 2xml 3bhb 3eyd 3ibn 3ml5 3q6s 3t8s 3zeb 4bw4 4fcm 4iwz 4ly1 4p0w 1f3j 1k3q 1owh 1ulg 2aeb 2g9x 2o7v 2uw7 2xmy 3bho 3eyf 3ibu 3mlb 3q6w 3t8v 3zep 4bxk 4fcq 4ixh 4ly9 4p0x 1f40 1k3t 1owi 1uml 2aei 2ga2 2o8h 2uw8 2xn3 3bhx 3eyg 3idp 3mle 3q6z 3t8w 3zev 4bxn 4fcr 4ixu 4lyn 4p10 1f47 1k4g 1owj 1umw 2ael 2gbf 2o9a 2uwd 2xn5 3bi0 3eyh 3ie3 3mmf 3q71 3t9t 3zh8 4bxu 4fe6 4ixv 4lys 4p1r 1f4e 1k4h 1owk 1ung 2aez 2gbg 2o9i 2uwl 2xn6 3bi1 3eyl 3iej 3mmr 3q72 3ta0 3zha 4byi 4fe9 4iz0 4lyw 4p1u 1f4f 1k6c 1ox9 1unh 2afw 2gbi 2o9j 2uwo 2xn7 3bi6 3eys 3ieo 3mn8 3q77 3ta1 3zhf 4byj 4fea 4izm 4lzr 4p2t 1f4g 1k6p 1oxg 1unl 2afx 2gc8 2o9k 2uwp 2xnb 3bim 3eyu 3ies 3mna 3q7j 3tam 3zhx 4bzn 4feq 4izy 4lzs 4p3h 1f4x 1k6t 1oxn 1uom 2agv 2gcd 2o9r 2uxu 2xne 3biz 3ezr 3iet 3mnu 3q7p 3tao 3zhz 4bzr 4fev 4j03 4m0e 4p4s 1f4y 1k6v 1oxq 1uou 2ai7 2gd8 2o9v 2uxx 2xng 3bjc 3ezv 3if7 3mo0 3q7q 3tay 3zi0 4bzs 4few 4j04 4m0f 4p4t 1f57 1k9q 1oxr 1upf 2ai8 2gde 2oa0 2uxz 2xni 3bjm 3f07 3ifl 3mo2 3q8d 3tb6 3zi8 4c0r 4ff8 4j06 4m0r 4p58 1f5k 1k9s 1oy7 1upk 2aia 2gdo 2oag 2uy0 2xnm 3bki 3f0r 3ifo 3mo5 3q8h 3tc5 3zim 4c1m 4ffs 4j08 4m0y 4p5d 1f5l 1ka7 1oyn 1ur9 2aie 2gej 2oah 2uy3 2xnn 3bkk 3f15 3ifp 3mo8 3q92 3tcg 3zj6 4c1t 4fgx 4j09 4m0z 4p5z 1f74 1kak 1oyq 1urc 2aig 2gek 2oax 2uy4 2xno 3bkl 3f16 3ig1 3moe 3q96 3tcp 3zj8 4c1u 4fgy 4j0a 4m12 4p6e 1f7b 1kat 1oyt 1urg 2aj8 2gfd 2oaz 2uy5 2xnp 3bl0 3f17 3ig6 3mof 3qa2 3tct 3zjc 4c1w 4fgz 4j0p 4m13 4p6g 1f8a 1kav 1oz0 1urw 2ajb 2gfj 2obf 2uyi 2xo8 3bl1 3f18 3ig7 3moh 3qaa 3tcy 3zju 4c2v 4fhh 4j0r 4m14 4p6w 1f8b 1kc5 1ozv 1usi 2ajd 2gfs 2obj 2uym 2xog 3bl2 3f19 3igb 3mp1 3qai 3td4 3zjv 4c35 4fhi 4j0s 4m1d 4p6x 1f8c 1kc7 1p01 1usk 2ajl 2gg0 2obo 2uyn 2xoi 3bl7 3f1a 3igg 3mp6 3qak 3tdc 3zk6 4c36 4fht 4j0t 4m1j 4p72 1f8d 1kcs 1p02 1usn 2al5 2gg2 2oc0 2uyq 2xow 3bl9 3f2a 3igp 3mpe 3qaq 3tdh 3zke 4c37 4fi9 4j0v 4m2u 4p73 1f8e 1kdk 1p03 1utc 2alv 2gg3 2oc1 2uyw 2xp2 3bla 3f33 3igv 3mpf 3qar 3tdj 3zkf 4c38 4fic 4j0y 4m2v 4p75 1f90 1kds 1p04 1uti 2am1 2gg5 2oc2 2uz6 2xp3 3blr 3f34 3ihz 3mpm 3qbc 3tdu 3zki 4c3k 4fiv 4j0z 4m2w 4p7e

3. PDB IDs for database B

1m5c 1z4n 2ves 3n1v 4gxs 1akt 1m5w 1zzl 2vti 3pce 4h58 1awh 1mqj 2aa6 2w87 3q2j 4iq6 1bzs 1mue 2ajd 2wbg 3qch 4j0t 1c9d 1n95 2bqv 2wi5 3r91 4j21 1d3p 1ndv 2bxu 2wkz

3s2p 4kao 1d4i 1nqc 2c93 2xp4 3t2q 4lko 1f4g 1o2j 2ccc 2zp0 3vry 4mr6 1f4y 1p6e 2hpa 3bc4 3vw1 4pzv 1flr 1pa9 2ilp 3c56 3zbx 1gi7 1ph0 2j7d 3ebl 3zlv 1hih 1s5z 2j83 3fr2 4a6c 1i48 1sme 2oaz 3gn7 4anv 1il3 1tnl 2ohmt 3gww 4auy 1il9 1trd 2p3d 3i6z 4bnu 1jil 1v48 2pj6 3ik3 4do4 1k03 1wb0 2pq9 3k41 4e5w 1k1j 1xn0 2rd6 3ljo 4eh4 1m2p 1xpc 2rfy 3mxd 4gja 1a8i 1pbk 2d0k 2wih 3l0v 4b73 1acj 1qb6 2dxs 2xj2 3l3m 4bcm 1ajn 1qbr 2eg7 2xtk 3l59 4bi6 1b6p 1rbo 2fda 2ypi 3l9l 4br3 1bl4 1re8 2fkf 2ywp 3lzv 4c4f 1bmm 1rm8 2flr 2zbk 3mg4 4dfg 1bqo 1rtf 2fm2 2zjf 3msl 4dgn 1bwa 1td7 2g0h 2zzu 3mv0 4eeh 1c70 1tsy 2gfs 3a6t 3n7a 4eft 1c87 1u3r 2gh9 3alt 3nef 4eoi 1cs4 1udt 2gtk 3ao2 3nm6 4eon 1d6v 1uho 2gu8 3as0 3np7 4f49 1drk 1utm 2h42 3bet 3nuu 4ght 1dzk 1uv6 2h5a 3bl9 3nyd 4gii 1e6q 1uwb 2i5f 3bug 3nzx 4gk2 1e9h 1uyk 2igx 3c45 3o57 4gzx 1err 1v2l 2jc0 3ccc 3o9b 4hup 1ezf 1vjy 2nmx 3cf8 3o9e 4hyu 1f8b 1w13 2nnd 3cgf 3oeu 4i73 1fl6 1w2k 2nnp 3clp 3oy3 4i74 1fv0 1w4o 2o3p 3cwk 3pd8 4i8w 1g37 1w4q 2o3z 3d1v 3pke 4inr 1gj5 1w5x 2obf 3d20 3pwm 4io2 1gzv 1w80 2ojf 3d7f 3qnd 4iva 1h2k 1wbt 2on3 3da9 3qtq 4ivk 1ha2 1ws4 2onc 3dc2 3qtv 4ixv 1hbj 1wzy 2ork 3dnd 3r7o 4j1c 1hms 1x6u 2piz 3ehy 3r7q 4j1i 1hpx 1x78 2pk5 3eid 3r8v 4j4o 1ibg 1xh4 2q2y 3ejr 3rsx 4j8t 1ii5 1xow 2qbu 3f78 3s0e 4jjs 1j17 1xuo 2qch 3f7h 3s0o 4k63 1j4r 1y2c 2qju 3fas 3sm2 4k69 1jd0 1y91 2qnn 3fcl 3t60 4k6i 1jmi 1yp9 2qnp 3fnm 3ti1 4k8s 1k22 1yq7 2qnz 3fxw 3ti6 4kby 1kr3 1yqy 2qyn 3g0b 3ttm 4kiq 1ktt 1z1h 2r2w 3g5d 3u10 4kqo 1kv1 1z6e 2r3j 3gcu 3u3u 4kyh 1lhd 1z6q 2r3t 3gta 3v3q 4l2g 1lhw 1zd5 2r5p 3gy7 3v7s 4l34 1lnm 1zom 2ra0 3h2f 3vhk 4ly9 1lpz 220l 2rgp 3h5u 3vhu 4mcb 1ml1 2aei 2rku 3hf8 3w07 4mcv 1n43 2ank 2sim 3hfb 3wd9 4mmf 1np0 2aov 2uw5 3hv5 3zc5 4pnt 1nw7 2b1p 2uw8 3i3b 3zrk 1o33 2bet 2v00 3ig6 4a9n 1o44 2bkz 2v77 3ipx 4aa5 1o4d 2bok 2vc9 3itc 4ahr 1o4p 2c6n 2vio 3ivh 4ai5 1oba 2cem 2viv 3jwq 4akn 1ofz 2ces 2vl4 3kaf 4alw 1oi9 2chx 2vw1 3kqa 4as9 1ouk 2csn 2w8j 3kvx 4axa 1owj 2w8y 3kyr 4azt
